# Mortality risk of antipsychotic augmentation for adult depression

**DOI:** 10.1371/journal.pone.0239206

**Published:** 2020-09-30

**Authors:** Tobias Gerhard, T. Scott Stroup, Christoph U. Correll, Soko Setoguchi, Brian L. Strom, Cecilia Huang, Zhiqiang Tan, Stephen Crystal, Mark Olfson

**Affiliations:** 1 Center for Pharmacoepidemiology and Treatment Science, Institute for Health, Health Care Policy and Aging Research; Rutgers University, New Brunswick, NJ, United States of America; 2 Department of Pharmacy Practice and Administration, Ernest Mario School of Pharmacy, Rutgers University, Piscataway, NJ, United States of America; 3 Department of Psychiatry, College of Physicians and Surgeons, Columbia University and the New York State Psychiatric Institute, New York, NY, United States of America; 4 Department of Psychiatry, The Zucker Hillside Hospital, Glen Oaks, NY, United States of America; 5 Department of Psychiatry and Molecular Psychiatry, Hofstra Northwell School of Medicine, Hempstead, NY, United States of America; 6 Department of Child and Adolescent Psychiatry, Charité Universitätsmedizin Berlin, Berlin, Germany; 7 Department of Statistics and Biostatistics, Rutgers University, Piscataway, NJ, United States of America; 8 Center for Health Services Research on Pharmacotherapy, Chronic Disease Management, and Outcomes, Institute for Health, Health Care Policy and Aging Research, Rutgers University, New Brunswick, NJ, United States of America; University of British Columbia, CANADA

## Abstract

**Importance:**

Randomized controlled trials have demonstrated increased all-cause mortality in elderly patients with dementia treated with newer antipsychotics. It is unknown whether this risk generalizes to non-elderly adults using newer antipsychotics as augmentation treatment for depression.

**Objective:**

This study examined all-cause mortality risk of newer antipsychotic augmentation for adult depression.

**Design:**

Population-based new-user/active comparator cohort study.

**Setting:**

National healthcare claims data from the US Medicaid program from 2001–2010 linked to the National Death Index.

**Participants:**

Non-elderly adults (25–64 years) diagnosed with depression who after ≥3 months of antidepressant monotherapy initiated either augmentation with a newer antipsychotic or with a second antidepressant. Patients with alternative indications for antipsychotic medications, such as schizophrenia, psychotic depression, or bipolar disorder, were excluded.

**Exposure:**

Augmentation treatment for depression with a newer antipsychotic or with a second antidepressant.

**Main outcome:**

All-cause mortality during study follow-up ascertained from the National Death Index.

**Results:**

The analytic cohort included 39,582 patients (female = 78.5%, mean age = 44.5 years) who initiated augmentation with a newer antipsychotic (n = 22,410; 40% = quetiapine, 21% = risperidone, 17% = aripiprazole, 16% = olanzapine) or with a second antidepressant (n = 17,172). The median chlorpromazine equivalent starting dose for all newer antipsychotics was 68mg/d, increasing to 100 mg/d during follow-up. Altogether, 153 patients died during 13,328 person-years of follow-up (newer antipsychotic augmentation: n = 105, follow-up = 7,601 person-years, mortality rate = 138.1/10,000 person-years; antidepressant augmentation: n = 48, follow-up = 5,727 person-years, mortality rate = 83.8/10,000 person-years). An adjusted hazard ratio of 1.45 (95% confidence interval, 1.02 to 2.06) indicated increased all-cause mortality risk for newer antipsychotic augmentation compared to antidepressant augmentation (risk difference = 37.7 (95%CI, 1.7 to 88.8) per 10,000 person-years). Results were robust across several sensitivity analyses.

**Conclusion:**

Augmentation with newer antipsychotics in non-elderly patients with depression was associated with increased mortality risk compared with adding a second antidepressant. Though these findings require replication and cannot prove causality, physicians managing adults with depression should be aware of this potential for increased mortality associated with newer antipsychotic augmentation.

## Introduction

Depressive disorders are a leading cause of disability and emotional, physical, and economic burden [1]. Antidepressants are the first-line pharmacological treatment option for major depressive disorder, but inadequate response is common, with more than half of patients not achieving remission from their first antidepressant trial [2]. Clinical approaches to incomplete response include switching to another antidepressant followed by various augmentation strategies [3, 4]. Although not widely supported by treatment guidelines, augmentation with concomitant antidepressants is common in US practice^4^ and has some empirical support [5–8]. Augmentation with newer antipsychotics was at the time of the study the only treatment option approved by the US Food and Drug Administration (aripiprazole, quetiapine, olanzapine/fluoxetine). Randomized controlled trials of augmentation with newer antipsychotics have demonstrated efficacy in reducing observer-rated depressive symptoms [9,10], but have been criticized for their methodology and lack of demonstration of benefit on quality of life and functional outcomes [11, 12].

Further, newer antipsychotics have well characterized serious adverse effects, including rapid weight gain, diabetes, and tardive dyskinesia [13, 14]. Newer antipsychotics have also been associated with increased mortality, most notably a >50% increased mortality risk in older adults with dementia [15, 16]. However, due to the restricted inclusion criteria and limited sample sizes of RCTs for newer antipsychotic augmentation [11, 17–19], these trials are uninformative with regard to a potential mortality risk for augmentation with newer antipsychotics for depression in clinical practice.

The safety of newer antipsychotic augmentation for depression should be viewed in the context of limited therapeutic benefits [11, 18] and widespread clinical use [20–23]. In the US, approximately 2 million office-based medical visits per year for depression include prescriptions for newer antipsychotics [20]. Furthermore, most patients who initiate newer antipsychotic treatment for nonpsychotic depression either do not have a prior adequate trial with antidepressants or have initiated the newer antipsychotic without a concomitant antidepressant [21]. These prescribing practices raise concern that newer antipsychotic initiation for depression may sometimes be premature or clinically inappropriate.

The uncertain safety profile of augmentation with newer antipsychotics for depression complicates clinical decisions concerning their appropriate role in treatment for patients who do not have an adequate response to antidepressant monotherapy. This study aimed to estimate the real-world mortality risk of newer antipsychotics in non-elderly adults with depression and an incomplete response to antidepressant monotherapy.

## Methods

### Data source and study cohort

The study cohort was assembled from the Medicaid Analytic eXtract (MAX) for 45 states from 2001–2010 [24]. Data from Arizona, Delaware, Nevada, Oregon, and Rhode Island were not available. MAX data include information on diagnoses of all paid inpatient, emergency, and outpatient services using standard ICD codes, as recorded by the treating provider and records of all paid claims for dispensed medications, including National Drug Codes, dispensing dates, and days and quantity of medications supplied [25]. Date and cause of death were identified by linkage to the National Death Index [26]. The study was approved by the Rutgers University Institutional Review Board.

The study compared all-cause and select cause-specific mortality between non-elderly adult (25–64 years) patients with depression who, after a 90-day period of stable antidepressant treatment, either initiated augmentation with a newer antipsychotic or with a second antidepressant (Fig 2). Initiation of augmentation treatment defined the index date and the beginning of follow-up. Eligibility criteria were assessed during the 180 days immediately preceding the index date. All cohort patients had uninterrupted Medicaid coverage during the 180-day pre-index period, ≥1 first listed inpatient or outpatient diagnosis of depression during the first 90 days of the pre-index period (ICD-9-CM 296.2, 296.3, 300.4, 311), and no break of >7 days in medication supply with a single antidepressant medication during the 90 days directly preceding the index date. For patients initiating a second antidepressant, we required that the initial antidepressant was refilled on the same day to maximize the likelihood that the clinical intent was to initiate augmentation with a second antidepressant rather than to switch between antidepressants.

Patients with alternative indications for newer antipsychotics including psychotic depression, schizophrenia, bipolar disorder, autism-spectrum disorders, or dementia were excluded. Patients were also excluded if they had any antipsychotic medication use (including older antipsychotics) during the baseline period, used more than one antidepressant medication during the 60 days preceding their index date, initiated a second antidepressant and an newer antipsychotic on their index date, or were diagnosed with a life threatening disorder [16].

### Exposure and follow-up time

All newer antipsychotics available in the U.S. during the study period, except clozapine, were included in the analysis (asenapine, aripiprazole, iloperidone, olanzapine, paliperidone, quetiapine, risperidone, ziprasidone). Although only some of these newer antipsychotics are FDA-approved for the adjunctive treatment of depression, all except for clozapine are used clinically for this indication [20, 21]. Antidepressants included selective serotonin reuptake inhibitors (SSRIs), serotonin noradrenaline reuptake inhibitors (SNRIs), atypical antidepressants, and other antidepressants (see eTable 1, S1 Appendix for a complete list of antidepressants). Each day of follow-up was classified according to probable use of each antidepressant and newer antipsychotic. Patients were classified as discontinuing their augmentation treatment when the index drug was not refilled for >14 days after the end of medication supply to account for late refills and stockpiling. To examine dose response for all newer antipsychotics combined, doses were classified in chlorpromazine equivalents [27].

### Outcomes

The primary outcome was death that occurred during study follow-up. In addition to all-cause mortality, we separately examined natural and unnatural deaths, as well as non-cancer deaths (eTable 2, S1 Appendix). Unnatural deaths were defined as those caused by external causes, and included unintentional, suicide, homicide, undetermined intent, and other injuries. Date and cause of death were identified by linkage to the National Death Index. All-cause mortality data from the National Death Index have demonstrated 100% specificity with sensitivity between 98% and 100% [26, 28].

### Confounding variables

We measured a comprehensive list of known and potential risk factors for mortality including socio-demographics, diagnostic history, medication history, health care utilization history, and geography (eTable 3, S1 Appendix). Confounding variables were measured during the 180-day period immediately preceding the index date.

### Statistical analysis

We first compared baseline characteristics among initiators of each augmentation strategy. Propensity score methods, specifically inverse probability of treatment weighting, were used to control for measured confounding [29, 30]. The propensity score, the predicted probability of initiating augmentation with an newer antipsychotic versus a second antidepressant, was calculated using a logistic regression model including all variables in eTable 3 (S1 Appendix). Propensity score distributions were trimmed at the 2.5^th^ and 97.5^th^ percentiles of the exposed and unexposed groups, respectively, to reduce potential bias from unmeasured confounding by excluding patients who were treated contrary to strong prediction [31]. This approach was pre-specified in the study protocol and was based on a concern that treatment choice contrary to strong prediction likely stems from unmeasured confounding factors (S2 Appendix). Covariate balance was compared using standardized differences for all baseline covariates between treatment groups in the initial study cohort and after inverse probability of treatment weighting in the propensity score trimmed analytic cohort.

Cox proportional hazards regressions were fit to model the effect of alternative augmentation strategies on all-cause and cause-specific mortality. Models were fit without adjustment; adjusted for age, sex, race/ethnicity and index year; and adjusted via inverse probability of treatment weighting with robust variance estimation [32]. Follow-up began from the day after augmentation initiation and was censored at date of death, discontinuation of augmentation therapy, loss of Medicaid eligibility, or 365 days after the index date, whichever came first. Fourteen days were added to the last day of follow-up for patients who discontinued either augmentation therapy to reduce potential bias from informative censoring if patients discontinued the drug because of adverse effects experienced shortly before death. Follow-up was capped at 365 days because by this point a great majority of patients were expected to have discontinued index treatment.

Analyses were stratified by age group (25 to 54 years vs. 55 to 64 years) and sex. In addition, we performed individual comparisons between antidepressant augmentation and the most commonly used newer antipsychotics and explored dose-response based on the initial dose of the newer antipsychotic.

Lastly, we approximated the absolute difference in the incidence of death between patients in both groups. The adjusted rate difference was approximated as I_AD_*(aHR−1) with aHR as the estimated adjusted hazard ratio and I_AD_ as the unadjusted incidence rate for patients in the antidepressant augmentation cohort with corresponding 95% CIs [33].

### Sensitivity analyses

We performed a series of sensitivity analyses to examine the robustness of our results to changes in design or assumptions: [1] Alternate follow-up specifications (180 days, all available days of follow-up) and intent-to-treat analyses (no censoring of patients for changes in the augmentation treatments) [2]. Addition of discontinuation of the initial antidepressant to the study’s censoring criteria [3]. Exclusion of all patients with mood stabilizer use during the baseline period (lithium, carbamazepine, lamotrigine, valproic acid/valproate, divalproex, acetazolamide, felbamate, gabapentin, lacosamide, levetiracetam, oxcarbazepine, pregabalin, topiramate, zonisamide). Although mood stabilizers are sometimes used to treat unipolar depression [34], their use may indicate undiagnosed bipolar symptoms [4]. Limiting the study period to augmentation episodes initiated after January 1, 2007, as prescribing practices may have changed following FDA approval of newer antipsychotic augmentation in 2007 [5]. Alternative propensity score approaches, including adjustment for propensity score deciles and 1:1 propensity score matching [35], and analysis of the untrimmed cohort [30]. To empirically evaluate the rationale for propensity score trimming, we performed a *post-hoc* analysis stratified across the distribution of the propensity score. To provide perspective regarding the robustness of the study results to unmeasured confounding, we estimated the strength of the residual confounding required to fully explain the observed associations for all-cause mortality if in fact no association existed [36].

All analyses were performed with SAS version 9.4 (SAS Institute, Cary, North Carolina). All *P*-values were 2-sided with a *P* value < .05 indicating statistical significance. The study used de-identified data and was approved with a waiver of informed consent by the Rutgers Institutional Review Board.

## Results

**Fig 1:**
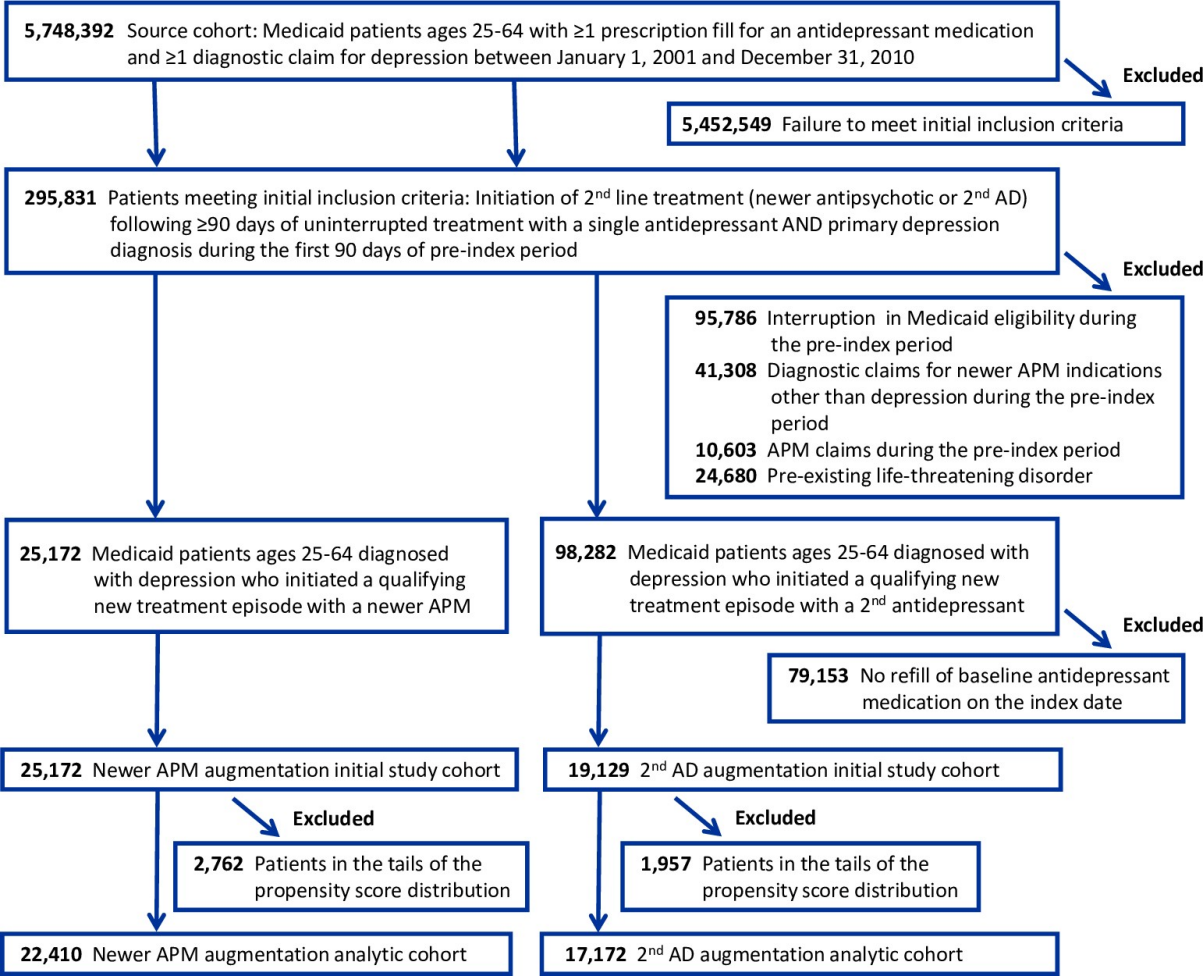
Flowchart of study cohort. AD, antidepressant; APM, antipsychotic medication.

The initial study cohort included 44,301 patients: 25,172 initiators of antipsychotic augmentation and 19,129 initiators of augmentation with a second antidepressant (Fig 1). Baseline antidepressants were SSRIs (56%), atypical antidepressants (20%), SNRIs (18%), and other antidepressants (6%), with minimal differences between treatment cohorts (eTable 1, S1 Appendix). In the antipsychotic augmentation cohort, quetiapine was the most commonly prescribed newer antipsychotic (40%), followed by risperidone (21%), aripiprazole (17%), and olanzapine (16%) (eTable 4, S1 Appendix). The remaining newer antipsychotics together made up the remaining 6%. The median chlorpromazine equivalent starting daily dose for all newer antipsychotics combined was 68 mg. During follow-up, the median combined chlorpromazine equivalent dose increased to 100 mg. Starting and final doses for commonly used individual antipsychotics (actual mg as well as mg chlorpromazine equivalents) are shown in eTable 4 (S1 Appendix). Add-on antidepressants in the comparator cohort were most commonly atypical antidepressants (59%), followed by SSRIs (21%), other antidepressants (11%), and SNRIs (9%; eTable 1, S1 Appendix).

Patients in the initial cohort were on average 44 years old, predominantly female (78%), non-Hispanic white (70%), and eligible for Medicaid due to disability (64%). The most prevalent comorbid baseline diagnoses were anxiety (25%), hypertension (23%), hyperlipidemia (15%), and diabetes (14%) (Table 1 and eTable 3, S1 Appendix). Over the 180-day baseline period, study patients averaged 9.2 outpatient visits for depression and 15.5 non-mental health outpatient visits. Seventy-five percent had prescription claims for psychotropic drugs other than antidepressants or antipsychotics, most commonly anxiolytics/hypnotics (64%) and mood stabilizers (32%). The two treatment groups were generally comparable in their baseline characteristics but showed meaningful differences as indicated by standardized differences of >10% for several potential confounding variables (Table 1 and eTable 3, S1 Appendix). After propensity score trimming and inverse probability of treatment weighting, group differences were markedly diminished (Table 1). The resulting analytic study cohort included 39,582 patients (78.5% female, mean age 44.5 years), 22,410 initiators of augmentation with a newer antipsychotic, and 17,172 initiators of a second antidepressant (Fig 1). Characteristics of individuals excluded from the analytic cohort due to propensity score trimming are shown in eTable 3 (S1 Appendix).

**Table 1:** Selected baseline characteristics for new initiators of study augmentation regimens.

	Initial Study Cohort N = 44,301			Analytic Cohort^a^ N = 39,582		
	Newer Antipsychotic n = 25,172	Antidepressant n = 19,129		Newer Antipsychotic n = 22,410	Antidepressant n = 17,172	
	%	%	Std. Dif.	%	%	Std. Dif.
Female sex	76.0	80.7	.114	78.5	78.5	.002
Age, years (mean)	44.2	44.4	.022	44.4	44.5	.013
Race/Ethnicity						
White, non-Hispanic	70.3	68.9	.030	69.2	68.9	.007
Black, non-Hispanic	9.1	8.1	.038	8.7	8.8	.001
Hispanic	9.8	10.7	.029	10.5	10.6	.003
Other	10.8	12.4	.048	11.6	11.8	.007
Medicaid Eligibility						
Disability	67.6	59.4	.170	63.6	64.0	.009
Low income	20.9	26.4	.132	23.5	23.1	.009
Other/unknown	11.6	14.2	.077	12.9	12.9	.001
Diagnostic History, past 180 days						
Anxiety	26.8	23.3	.079	25.2	25.3	.005
Substance use disorder	7.8	6.2	.066	6.8	6.7	.004
Diabetes	14.2	13.3	.025	13.8	13.7	.001
Hyperlipidemia	14.7	15.5	.022	15.2	15.2	.003
Anemia	5.2	4.9	.014	5.1	5.1	.003
Hypertension	23.0	23.7	.017	23.3	23.4	.001
Ischemic heart disease	4.8	4.8	.000	4.9	4.8	.002
Cardiac dysrhythmias	2.8	2.5	.017	2.7	2.6	.004
Heart failure	2.0	1.7	.020	1.9	1.9	.001
Cerebrovascular disease	3.2	2.5	.042	2.8	2.7	.005
Medication History, past 180 days						
Psychotropic medication	79.4	70.3	.210	76.8	77.0	.004
Mood Stabilizer	36.9	25.0	.261	30.7	30.7	.001
Anxiolytic/hypnotics	67.1	60.7	.132	65.5	65.8	.006
Metabolic and related medication	30.1	28.2	.042	29.3	29.6	.007
Cardiovascular medication	40.1	39.8	.006	40.2	40.2	.002
Respiratory/allergy medication	54.1	52.1	.040	53.2	53.5	.006
Antibiotics	52.0	50.7	.026	51.2	51.4	.004
Diabetic medication	13.4	12.5	.024	13.1	13.1	.000
Hyperlipidemia medication	23.2	22.0	.029	22.9	23.2	.007
Acute services, past 180 days						
Non-MH hospitalization	8.5	7.2	.047	7.9	7.5	.016
MH hospital admission	5.3	2.9	.121	3.3	3.3	.000
OP visits for depression (mean)	10.1	8.0	.148	8.5	8.4	.011
Non-MH outpatient visits (mean)	16.3	14.4	.104	14.8	14.8	.000
MH outpatient visits (mean)	13.9	10.1	.199	10.5	10.3	.014

^a^After propensity score trimming and inverse probability of treatment (IPT) weighting; Newer Antipsychotic denotes the cohort initiating augmentation treatment with a newer antipsychotic; Antidepressant denotes the cohort initiating augmentation treatment with a second antidepressant medication; Std. Dif., standardized difference

In the analytic cohort, initiators of newer antipsychotics had 105 deaths during 7,601 person-years of follow-up (138.1 per 10,000 person-years) (Table 2). Initiators of antidepressant augmentation had 48 deaths during 5,727 person-years of follow-up (83.8 per 10,000 person-years). Discontinuation of the index treatment was the most common reason for censoring (82.6%), followed by day 365 (10.2%), end of the study period (3.4%), loss of Medicaid eligibility (3.3%), and occurrence of the outcome event (0.4%). There were minimal group differences in reasons for censoring. The adjusted hazard ratio for all-cause mortality comparing newer antipsychotic to antidepressant augmentation was 1.45 (95% confidence interval (CI), 1.02–2.06), with a risk difference of 37.7 (95%CI, 1.7–88.8) per 10,000 person-years. The adjusted Kaplan-Meier plot is shown in eFig 1 (S1 Appendix). No dose-response effect was apparent (eTable 5, S1 Appendix).

**Table 2:** Mortality according to underlying cause of death, N = 39,582^a^.

	Newer Antipsychotic Augmentation^b^		Antidepressant Augmentation^c^			
Deaths	Deaths	Incidence per 10,000 Person-Years	Deaths	Incidence per 10,000 Person-Years	Adjusted Hazard Ratio (95% CI)	Adjusted Rate Difference (per 10,000 years of follow-up) (95% CI)
All^d^	105	138.1	48	83.8	1.45 (1.02 to 2.06)	37.7 (1.7 to 88.8)
Natural	69	90.8	30	52.4	1.58 (1.02 to 2.45)	30.4 (1.0 to 76.0)
Non-Cancer	68	89.5	28	48.9	1.65 (1.05 to 2.60)	31.8 (2.4 to 78.2)
Unnatural	29	38.2	14	24.4	1.21 (0.63 to 2.34)	5.1 (-9.0 to 32.7)

^a^After propensity score trimming and inverse probability of treatment weighting

^b^7,601 person years of follow-up

^c^5,727 person years of follow-up

^d^includes 11 deaths with unknown or missing cause of death.

Estimates were consistent when the endpoint was restricted to natural death (HR = 1.58, 95%CI 1.02–2.45) or non-cancer death (HR = 1.65, 95%CI 1.05–2.60). Risk for unnatural death showed a modest numerical increase but confidence intervals were consistent with both harmful and protective effects (HR = 1.21, 95%CI 0.63–2.34).

### Stratified analyses

Analyses stratified by age group and sex and for individual newer antipsychotics are shown in Table 3. Newer antipsychotics were associated with a mortality risk of HR = 1.61 (95%CI 0.92–2.80) in older adult patients and a mortality risk of HR = 1.36 (95%CI 0.86–2.13) in younger adult patients. Newer antipsychotics showed an association with increased mortality risk among women (HR = 1.72, 95%CI 1.13–2.63), but not among men (HR = 0.99, 95%CI 0.52–1.87).

**Table 3:** All-cause mortality by age group, sex, and individual newer antipsychotic medication, N = 39,582^a^.

	Newer Antipsychotic Augmentation		Antidepressant Augmentation			
Subgroup	Deaths	Person Years	Deaths	Person Years	Adjusted Hazard Ratio (95% CI)	Adjusted Rate Difference (per 10,000 years of follow-up) (95% CI)
*Age Group*						
25 to 54	64	5,988	29	4,424	1.36 (0.86 to 2.13)	23.6 (-9.2 to 74.1)
55 to 64	41	1,613	19	1,303	1.61 (0.92 to 2.80)	88.9 (-11.7 to 262.5)
*Sex*						
Female	77	5,716	32	4,533	1.72 (1.13 to 2.63)	50.8 (9.2 to 115.1)
Male	28	1,885	16	1,194	0.99 (0.52 to 1.87)	-1.3 (-64.3 to 116.6)
*Generic APM*						
Quetiapine	38	3,021	51	5,600	1.18 (0.77 to 1.82)	16.4 (-20.9 to 74.7)
Risperidone	25	1,568	50	5,669	1.66 (1.01 to 2.74)	58.2 (0.9 to 153.5)
Aripiprazole	11	1,183	31	3,678	0.88 (0.42 to 1.86)	-10.1 (-48.9 to 72.5)
Olanzapine	22	1,155	44	5,387	1.92 (1.10 to 3.33)	75.1 (8.2 to 190.3)

^a^After propensity score trimming and inverse probability of treatment weighting; 365 day maximum follow-up, as-treated

### Analyses of individual newer antipsychotics

When augmentation with individual newer antipsychotics was compared to augmentation with a second antidepressant, olanzapine showed the greatest increase in risk (HR = 1.92, 95%CI 1.10–3.33), followed by risperidone (HR = 1.66, 95%CI 1.01–2.74), quetiapine (HR = 1.18, 95%CI 0.77–1.82), and aripiprazole (HR = 0.88, 95%CI 0.42–1.86). Due to sample size limitations, stratified analyses and analyses of individual newer antipsychotics were considered exploratory and no formal interaction tests were performed.

### Sensitivity analyses

Sensitivity analyses related to follow-up period, study period, and propensity score implementation, yielded consistent findings (Table 4). Adding discontinuation of the initial antidepressant to the censoring criteria for both treatment groups resulted in a loss of approximately 22% of follow-up time but did not meaningfully alter the results for all-cause mortality (HR = 1.47, 95%CI 0.95–2.28). Exclusion of patients with claims for mood stabilizers during the baseline period numerically increased the hazard ratio for all-cause mortality to 1.70 (95%CI 1.09–2.65). Results were sensitive to the implementation of propensity score trimming. In the untrimmed cohort, point estimates of the mortality hazard ratio moved towards the null hypothesis and hazard ratio confidence intervals crossed 1.0 (Table 4). Propensity score stratified analysis indicated homogenous mortality risk in the central propensity score strata but strong heterogeneity in the trimmed tails of the propensity score (eTable 6, S1 Appendix). Unadjusted results for all inferential analyses are shown in eTables 7–9 (S1 Appendix).

**Table 4:** Sensitivity analyses, N = 39,582.

	Newer Antipsychotic Augmentation		Antidepressant Augmentation			
	Deaths	Person Years	Deaths	Person Years	Adjusted Hazard Ratio (95% CI)	Adjusted Rate Difference (per 10,000 years of follow-up) (95% CI)
*Follow-up specification*^a^						
365-day follow-up As-Treated	105	7,601	48	5,727	1.45 (1.02 to 2.06)	37.7 (1.7 to 88.8)
365-day follow-up ITT	264	19,798	135	15,204	1.26 (1.02 to 1.56)	23.1 (1.8 to 49.7)
180-day follow-up As-Treated	79	5,848	35	4,442	1.47 (0.98 to 2.22)	37.0 (-1.6 to 96.1)
180-day follow-up ITT	142	10,400	64	7,986	1.40 (1.04 to 1.90)	32.1 (3.2 to 72.1)
All days in study period As-Treated	137	9,916	71	7,289	1.26 (0.94 to 1.69)	25.3 (-5.8 to 67.2)
Censored at baseline antidepressant discontinuation	72	5,872	31	4,463	1.47 (0.95 to 2.28)	32.6 (-3.5 to 88.9)
*Exclusion criteria*^*a*^						
Excluding mood stabilizer use during baseline period	68	4,987	29	4,179	1.70 (1.09 to 2.65)	48.6 (6.2 to 114.5)
*Index Year* ^a^						
2007–2010	37	2,540	11	1,767	1.97 (0.98 to 3.94)	66.0 (2.5 to 191.1)
*PS Analysis*^*b*^						
PS decile-adjusted, trimmed	105	7,601	48	5,727	1.47 (1.04 to 2.08)	39.4 (3.4 to 90.5)
1:1 PS matched *(N = 33*,*502)*	74	5,669	54	5,583	1.35 (0.95 to 1.91)	33.9 (-4.8 to 88.0)
*PS Trimming*^*b*^						
Untrimmed, IPT-weighted *(N = 44*,*301)*	120	8,641	57	6,351	1.21 (0.87 to 1.69)	18.8 (-11.7 to 61.9)
Untrimmed, PS decile-adjusted *(N = 44*,*301)*	120	8,641	57	6,351	1.31 (0.95 to 1.81)	27.8 (-4.5 to 72.7)

^a^After propensity score trimming and inverse probability of treatment weighting

^b^365 day maximum follow-up, as-treated; PS, propensity score; IPT, inverse probability of treatment

Analyses adjusted for age, sex, race/ethnicity, and index year were generally consistent with inverse probability of treatment-weighted results (eTable 10, S1 Appendix). A relative risk of approximately 3.8 linking a hypothetical confounder in 25% of the population to both newer antipsychotic use and mortality would be needed to fully explain the observed association in the primary analysis (eFig 2, S1 Appendix).

## Discussion

Our study examined the mortality risk of newer antipsychotic augmentation for non-elderly adults with depression in a large cohort of US Medicaid insured patients. As compared to augmentation with an antidepressant, augmentation with a newer antipsychotic was associated with a 45% relative increase in mortality risk, equivalent, in our study population, to an absolute risk difference of 37.7 deaths per 10,000 person-years of treatment (0.38%/year). The magnitude of the observed relative increase in mortality risk is broadly similar to the findings of a meta-analysis of placebo-controlled randomized controlled trials for newer antipsychotics in elderly dementia patients (54%) [15], a finding that triggered a class-wide black box warning for newer antipsychotics by the US Food and Drug Administration [37].

Although the absolute mortality risk in patients with depression is markedly lower than in elderly patients with dementia, the magnitude of excess risk in the non-elderly depression group is substantial and warrants careful clinical consideration. A mortality rate difference of 37.7 per 10,000 years of follow-up corresponds to a number-needed-to-harm of approximately 265 per year. For higher risk subgroups the number-needed-to-harm decreases substantially, e.g., to 112 for patients between 55 and 64 years of age. Because of the small-to-moderate-size benefits of newer antipsychotic augmentation (meta analyses of newer antipsychotic augmentation RCTs estimate a number-needed-to-treat for remission of about 9) and lack of demonstrated benefit with regards to quality of life or functional impairment [11, 18], the additional mortality risk associated with newer antipsychotics is of great clinical relevance. This is particularly the case for higher risk subgroups, and especially considering that nearly two-thirds of patients initiating newer antipsychotics for depression in the United States do not first receive a full antidepressant trial [21].

Stratified analyses suggest potential clinically relevant heterogeneity by age, sex and choice of newer antipsychotic, with particularly high absolute risk differences in patients 55 to 64 years of age, women, and patients treated with olanzapine or risperidone. Importantly, the study’s power to examine these subgroups was limited and stratified results should be considered as hypothesis generating until refuted or confirmed by future research. Newer antipsychotic augmentation showed an association with increased risk for mortality in women but not in men. Although this finding may reflect chance, prior reports have suggested several adverse effects of antipsychotic medications, including weight gain, diabetes, and cardiovascular death may disproportionately affect women [38]. Further study is required to place the sex-stratified findings in context and elucidate potential mechanisms. There were also marked risk differences between individual newer antipsychotics. A higher mortality risk for olanzapine and risperidone than for quetiapine and aripiprazole aligns with studies in elderly patients with and without dementia [39–41], and with late life bipolar disorder [42], but findings require independent refutation or confirmation.

When we restricted the analyses to mortality from natural causes or to non-cancer mortality, the point estimates increased and remained statistically significant. The estimate for unnatural death was numerically small and not statistically significant. Sample size limitations prevented examination of specific causes of death. In contrast to some prior studies in older adults [39, 40], we found no strong evidence of a dose-response. However, these analyses used chlorpromazine equivalents, which are based on expert opinion rather than scientifically validated methodology, and were solely based on the initial newer antipsychotic dose ignoring dose titration over follow-up, and thus unable to identify anything other than a substantial dose-response effect.

**Fig 2:**
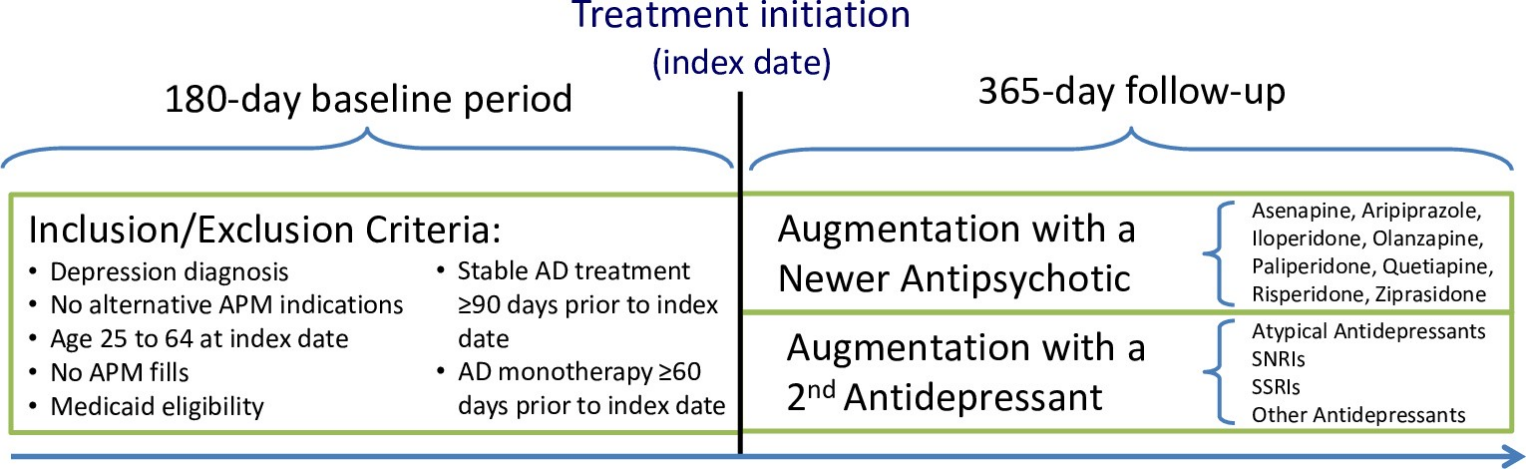
Study population and design. AD, antidepressant; atypical antidepressants (bupropion, mirtazapine, nefazodone, trazodone); SNRIs, serotonin noradrenalin reuptake inhibitors (desvenlafaxine, duloxetine, milnacipran, venlafaxine); SSRIs, selective serotonin reuptake inhibitors (including citalopram, escitalopram, fluoxetine, fluvoxamine, paroxetine, sertraline); other antidepressants (amitriptyline, amoxapine, clomipramine, desipramine, doxepin, imipramine, isocarboxazid, maprotiline, nortriptyline, protriptyline, tranylcypromine, trimipramine).

We selected augmentation treatment with a second antidepressant as the comparator group for this study as a means of controlling by study design for confounding by indication (Fig 2). This approach assures that both treatment groups are solely comprised of patients who initiated one of two augmentation treatments after at least 60 days on antidepressant monotherapy (a proxy measure for inadequate response to the baseline treatment). Although augmentation with a second antidepressant does not constitute a FDA-approved treatment strategy, it is widely used in clinical practice and the most suitable active comparator for our study. The consequence is that our results are relative to this treatment alternative. In other words, it is possible that we overestimated the mortality risk of newer antipsychotic augmentation (in case antidepressant augmentation reduces mortality risk) or that we underestimated the true mortality risk of newer antipsychotic augmentation (in case antidepressant augmentation increases mortality risk). However, to our knowledge there is currently no evidence to suggest that antidepressant augmentation affects mortality risk in patients with treatment resistant depression.

Our study had some limitations. First, as a nonrandomized study, residual confounding by factors associated with both newer antipsychotic use and increased risk of death, such as unmeasured psychotic symptoms, cannot be completely excluded. However, quantitative sensitivity analysis demonstrates that a strong and prevalent unmeasured confounder would be needed to fully explain the observed association. Second, study results were sensitive to the protocol-specified implementation of propensity score trimming, which excluded a small proportion of the study population in the tails of the propensity score distribution due to concerns for unmeasured confounding. However, this approach was supported by the strong heterogeneity of newer antipsychotic-associated mortality risk in the extremes of the propensity score distribution (eTable 6, S1 Appendix). Third, the limited number of deaths did not allow a more detailed examination of cause-specific mortality that may have provided insight into biologic mechanisms. Fourth, although the inclusion criteria required a refill of the initial antidepressant on the day of the index fill of the second antidepressant to maximize the likelihood that the intent was augmentation with a second antidepressant, some patients may have initiated the second antidepressant with the intent to switch antidepressants, which could capture patients with a somewhat lower severity of depression in the antidepressant augmentation group than the antipsychotic augmentation group. Finally, the results, which are confined to the US Medicaid population, may not generalize to other populations of non-elderly adults who are treated for depression.

## Conclusions

Our study suggests that augmentation with newer antipsychotics for depression may carry a mortality risk. These results warrant replication, ideally with a carefully designed publicly financed pragmatic RCT. The findings support careful consideration of this risk in relation to the limited known benefits of newer antipsychotics as adjuvants in treatment-resistant adult depression. The results further suggest use of newer antipsychotics only after non-response to evidence-based treatment options that are less risky.

## Supporting information

S1 Appendix eFig 1: Adjusted Kaplan-Meier Plot for All-cause Mortality. eFig 2: Sensitivity Analysis of Residual Confounding. eTable 1: Baseline and Add-on Antidepressants (Initial Study Cohort). eTable 2: ICD-10 Codes for Selected Causes of Death. eTable 3: Baseline Characteristics for New Initiators of Study Augmentation Regimens. eTable 4: Newer Antipsychotic Dose by Generic. eTable 5: Dose-Response for All-Cause Mortality. eTable 6: All-cause Mortality Stratified by Percentiles of the Propensity Score. eTable 7: Mortality According to Underlying Cause of Death (Untrimmed Cohorts, Unadjusted). eTable 8: All-cause Mortality by Age Group, Sex, and Individual Newer Antipsychotic Medication (Untrimmed Cohorts, Unadjusted). eTable 9: Sensitivity Analyses (Untrimmed Cohorts, Unadjusted). eTable 10: Mortality According to Underlying Cause of Death (Untrimmed Cohorts, Adjusted for Age, Sex, Race/Ethnicity and Index Year).
(DOCX)

S2 Appendix Study protocol (NIMH_R21MH102724). (PDF)
