# Supplementary material for: Mortality risk of antipsychotic augmentation for adult depression
Source: PLoS One. 2020 Sep 30;15(9):e0239206. doi: 10.1371/journal.pone.0239206 (PMC7526884; doi:10.1371/journal.pone.0239206)
Supplement: S1 Appendix — eFig 2: Sensitivity Analysis of Residual Confounding. eTable 1: Baseline and Add-on Antidepressants (Initial Study Cohort). eTable 2: ICD-10 Codes for Selected Causes of Death. eTable 3: Baseline Characteristics for New Initiators of Study Augmentation Regimens. eTable 4: Newer Antipsychotic Dose by Generic. eTable 5: Dose-Response for All-Cause Mortality. eTable 6: All-cause Mortality Stratified by Percentiles of the Propensity Score. eTable 7: Mortality According to Underlying Cause of Death (Untrimmed Cohorts, Unadjusted). eTable 8: All-cause Mortality by Age Group, Sex, and Individual Newer Antipsychotic Medication (Untrimmed Cohorts, Unadjusted). eTable 9: Sensitivity Analyses (Untrimmed Cohorts, Unadjusted). eTable 10: Mortality According to Underlying Cause of Death (Untrimmed Cohorts, Adjusted for Age, Sex, Race/Ethnicity and Index Year). (DOCX) [file pone.0239206.s001.docx]

**S1 Appendix (Supplementary Web Appendix)**

**Mortality Risk of Antipsychotic Augmentation for Adult Depression**

**Table of Contents**

**Page**

**eFigure 1: Adjusted Kaplan-Meier Plot for All-cause Mortality 2**

**eFigure 2: Sensitivity Analysis of Residual Confounding 3**

**eTable 1: Baseline and Add-on Antidepressants (Initial Study Cohort) 4**

**eTable 2: ICD-10 Codes for Selected Causes of Death 5**

**eTable 3: Baseline Characteristics for New Initiators of Study Augmentation Regimens 6**

**eTable 4: Newer Antipsychotic Dose by Generic 9**

**eTable 5: Dose-Response for All-Cause Mortality 10**

**eTable 6: All-cause Mortality Stratified by Percentiles of the Propensity Score 11**

**eTable 7: Mortality According to Underlying Cause of Death (Untrimmed Cohorts, Unadjusted) 12**

**eTable 8: All-cause Mortality by Age Group, Sex, and Individual Newer Antipsychotic 13**

**Medication (Untrimmed Cohorts, Unadjusted)**

**eTable 9: Sensitivity Analyses (Untrimmed Cohorts, Unadjusted) 14**

**eTable 10: Mortality According to Underlying Cause of Death (Untrimmed Cohorts, 15
Adjusted for Age, Sex, Race/Ethnicity and Index Year)**

**eFigure 1: Adjusted* Kaplan-Meier Plot for All-cause Mortality**


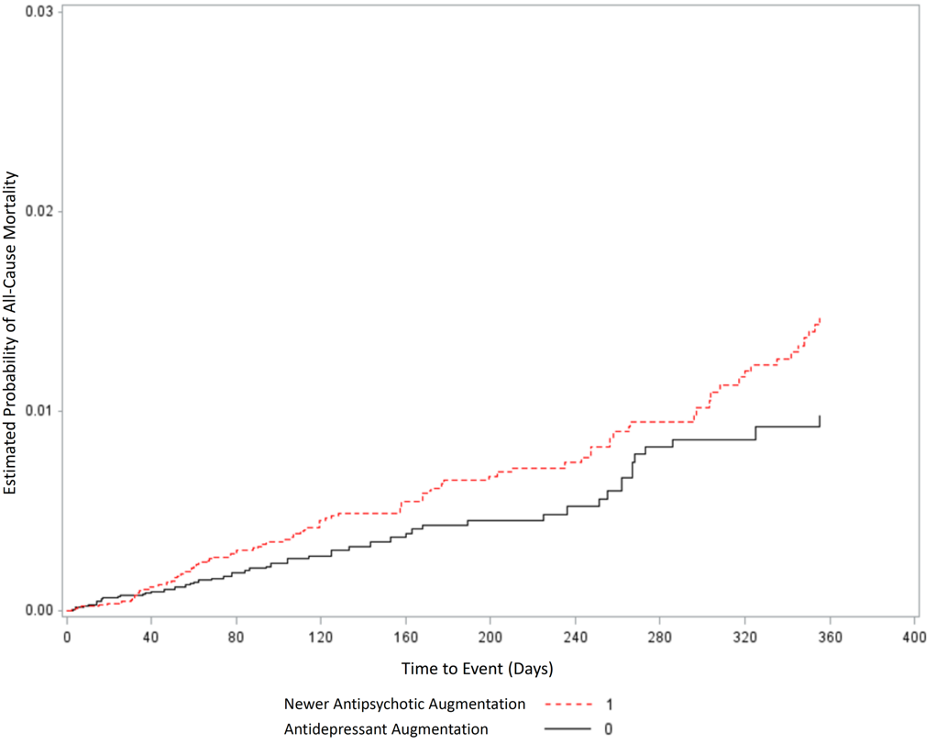


*Observations are inverse probability of treatment weighted. Log Rank p=0.036.

**eFigure 2: Sensitivity Analysis of Residual Confounding (Rule-out Approach): Example for Observed RR=1.45 and Different Levels of Confounder Prevalence (Pc=0.05; Pc=0.10; Pc=0.25)**

Each line splits the area into two. The upper right area represents all combinations of OREC and RRCD that would create confounding by an unmeasured factor strong enough to move the observed point estimate of the RR to the null (RR=1.0) or beyond. The area to the lower left represents all parameter combinations that would not be able to move the observed RR to the null.

OREC: Odds ratio between treatment status and presence of an unmeasured confounder.

RRCD: Relative Risk between an unmeasured confounder and the study outcome (all-cause mortality).

**eTable 1: Baseline and Add-on Antidepressants (Initial Study Cohort)**

|  | **Baseline Antidepressant  (Full Initial Study Cohort) N=44,301** | | **Baseline Antidepressant (Newer Antipsychotic Cohort) N=25,172** | | **Baseline Antidepressant (Antidepressant Cohort) N=19,129** | | **Add-on Antidepressant (Antidepressant Cohort) N=19,129** | |
| --- | --- | --- | --- | --- | --- | --- | --- | --- |
|  | n | % | n | % | n | % | n | % |
| **Atypical Antidepressants** | 8,730 | **19.71** | 4,800 | **19.07** | 3,930 | **20.54** | 11,218 | **58.64** |
| Bupropion | 3,658 | 8.26 | 1,962 | 7.79 | 1,696 | 8.87 | 3,792 | 19.82 |
| Mirtazapine | 2,202 | 4.97 | 1,264 | 5.02 | 938 | 4.90 | 1,674 | 8.75 |
| Nefazodone | 574 | 1.30 | 296 | 1.18 | 278 | 1.45 | 141 | 0.74 |
| Trazodone | 2,296 | 5.18 | 1,278 | 5.08 | 1,018 | 5.32 | 5,611 | 29.33 |
|  |  |  |  |  |  |  |  |  |
| **SSRIs** | 24,935 | **56.29** | 14,224 | **56.50** | 10,711 | **55.99** | 4,103 | **21.44** |
| Citalopram | 3,851 | 8.69 | 2,125 | 8.44 | 1,726 | 9.02 | 789 | 4.12 |
| Escitalopram | 4,340 | 9.80 | 2,650 | 10.53 | 1,690 | 8.83 | 1,049 | 5.48 |
| Fluoxetine | 5,301 | 11.97 | 3,045 | 12.10 | 2,256 | 11.79 | 723 | 3.78 |
| Fluvoxamine | 225 | 0.51 | 164 | 0.65 | 61 | 0.32 | 63 | 0.33 |
| Paroxetine | 5,379 | 12.14 | 2,906 | 11.54 | 2,473 | 12.93 | 654 | 3.42 |
| Sertraline | 5,839 | 13.18 | 3,334 | 13.24 | 2,505 | 13.10 | 825 | 4.31 |
|  |  |  |  |  |  |  |  |  |
| **SNRIs** | 8,102 | **18.28** | 4,774 | **19.32** | 3,328 | **17.40** | 1,749 | **9.15** |
| Desvenlafaxine | 227 | 0.51 | 160 | 0.64 | 67 | 0.35 | 45 | 0.24 |
| Duloxetine | 2,436 | 5.50 | 1,577 | 6.26 | 859 | 4.49 | 598 | 3.13 |
| Milnacipran | 10 | 0.02 | 6 | 0.02 | 4 | 0.02 | 21 | 0.11 |
| Venlafaxine | 5,429 | 12.25 | 3,031 | 12.40 | 2,398 | 12.54 | 1,085 | 5.67 |
|  |  |  |  |  |  |  |  |  |
| **Other Antidepressants^a^** | 2,534 | **5.71** | 1,374 | **5.44** | 1,160 | **6.08** | 2,059 | **10.77** |
| Amitriptyline | 1,330 | 3.00 | 724 | 2.88 | 606 | 3.17 | 1,156 | 6.04 |
| Amoxapine | 10 | 0.02 | 7 | 0.03 | 3 | 0.02 | 0 | 0 |
| Clomipramine | 93 | 0.21 | 61 | 0.24 | 32 | 0.17 | 37 | 0.19 |
| Desipramine | 79 | 0.18 | 39 | 0.15 | 40 | 0.21 | 57 | 0.30 |
| Doxepin | 415 | 0.94 | 226 | 0.90 | 189 | 0.99 | 346 | 1.81 |
| Imipramine | 214 | 0.48 | 109 | 0.43 | 105 | 0.55 | 134 | 0.70 |
| Isocarboxazid | 1 | 0.00 | 1 | 0.00 | 0 | 0 | 0 | 0 |
| Maprotiline | 3 | 0.01 | 2 | 0.01 | 1 | 0.01 | 1 | 0.01 |
| Nortriptyline | 358 | 0.81 | 185 | 0.73 | 173 | 0.90 | 308 | 1.61 |
| Protriptyline | 10 | 0.02 | 6 | 0.02 | 4 | 0.02 | 11 | 0.06 |
| Tranylcypromine | 11 | 0.02 | 11 | 0.04 | 0 | 0 | 1 | 0.01 |
| Trimipramine | 10 | 0.02 | 3 | 0.01 | 7 | 0.04 | 8 | 0.04 |

SSRIs, selective serotonin reuptake inhibitors; SNRIs, serotonin norepinephrine reuptake inhibitors; ^a^Other Antidepressants include tricyclic antidepressants, tetracyclic antidepressants, and monoaminoxidase inhibitors

**eTable 2: ICD-10 Codes for Selected Causes of Death**

| **Cause of Death** | **ICD-10 Code** |
| --- | --- |
| All causes | A00 - Y99 |
| Natural | A00 - R99 |
| Non-Cancer | A00 - B99, D00 - R99 |
| Un-Natural | U01-U03, V00-Y99 |

**eTable 3: Baseline Characteristics for New Initiators of Study Augmentation Regimens**

|  | **Initial Study Cohort**  **N=44,301** | | | **Analytic Cohort^a^ N=39,582** | | | **Individuals Excluded by Propensity Score Trimming**  **N=4,719** | |
| --- | --- | --- | --- | --- | --- | --- | --- | --- |
|  | **Newer APM**  **n=25,172** | **AD**  **n=19,129** |  | **Newer APM**  **n=22,410** | **AD**  **n=17,172** |  | **Newer APM**  **n=2,762** | **AD**  **n=1,957** |
|  | **%** | **%** | **Std. Dif.** | **%** | **%** | **Std. Dif.** | **%** | **%** |
| Sex |  |  |  |  |  |  |  |  |
| Male | 24.0 | 19.3 | .114 | 21.5 | 21.5 | .002 | 28.0 | 28.4 |
| Female | 76.0 | 80.7 | .114 | 78.5 | 78.5 | .002 | 72.0 | 71.6 |
|  |  |  |  |  |  |  |  |  |
| Age, years(mean) | 44.2 | 44.4 | .022 | 44.4 | 44.5 | .013 | 43.7 | 43.3 |
| 25-34 | 21.0 | 21.2 | .005 | 20.8 | 20.4 | .008 | 22.3 | 23.6 |
| 35-44 | 29.9 | 28.8 | .023 | 29.0 | 29.1 | .001 | 32.0 | 30.2 |
| 45-54 | 30.6 | 29.6 | .021 | 30.6 | 30.6 | .001 | 27.9 | 29.9 |
| 55-64 | 18.5 | 20.4 | .046 | 19.6 | 19.9 | .006 | 17.9 | 16.3 |
|  |  |  |  |  |  |  |  |  |
| Race/Ethnicity |  |  |  |  |  |  |  |  |
| White, non-Hispanic | 70.3 | 68.9 | .030 | 69.2 | 68.9 | .007 | 71.7 | 74.2 |
| Black, non-Hispanic | 9.1 | 8.1 | .038 | 8.7 | 8.8 | .001 | 8.6 | 9.0 |
| Hispanic | 9.8 | 10.7 | .029 | 10.5 | 10.6 | .003 | 8.3 | 7.6 |
| Other | 10.8 | 12.4 | .048 | 11.6 | 11.8 | .007 | 11.4 | 9.1 |
|  |  |  |  |  |  |  |  |  |
| Medicaid Eligibility |  |  |  |  |  |  |  |  |
| Disability | 67.6 | 59.4 | .170 | 63.6 | 64.0 | .009 | 69.1 | 65.4 |
| Low income | 20.9 | 26.4 | .132 | 23.5 | 23.1 | .009 | 19.6 | 23.3 |
| Other | 11.6 | 14.2 | .077 | 12.9 | 12.9 | .001 | 11.3 | 11.3 |
|  |  |  |  |  |  |  |  |  |
| Medicare Eligibility (Dual) | 20.0 | 16.2 | .149 | 18.9 | 19.1 | .006 | 25.3 | 22.2 |
| Managed Care | 25.4 | 28.1 | .061 | 27.3 | 27.3 | .001 | 21.7 | 22.5 |
|  |  |  |  |  |  |  |  |  |
| Augmentation Initiation year |  |  |  |  |  |  |  |  |
| 2001 | 4.9 | 8.1 | .128 | 4.9 | 4.9 | .001 | 17.4 | 16.6 |
| 2002 | 12.9 | 15.1 | .066 | 13.7 | 13.8 | .003 | 15.8 | 14.3 |
| 2003 | 13.0 | 13.3 | .010 | 13.1 | 13.1 | .000 | 12.4 | 11.0 |
| 2004 | 13.5 | 12.8 | .021 | 13.3 | 13.5 | .004 | 11.9 | 10.3 |
| 2005 | 12.2 | 11.0 | .038 | 11.7 | 11.8 | .002 | 10.9 | 11.4 |
| 2006 | 7.2 | 7.9 | .028 | 7.7 | 7.6 | .003 | 5.4 | 6.2 |
| 2007 | 7.1 | 7.8 | .024 | 7.7 | 7.7 | .000 | 5.2 | 5.7 |
| 2008 | 8.1 | 7.2 | .031 | 8.0 | 8.0 | .001 | 5.1 | 5.8 |
| 2009 | 10.1 | 7.9 | .077 | 9.3 | 9.2 | .004 | 8.2 | 10.1 |
| 2010 | 11.2 | 8.9 | .074 | 10.6 | 10.5 | .001 | 7.7 | 8.6 |
|  |  |  |  |  |  |  |  |  |
| Diagnostic History, past 180 days |  |  |  |  |  |  |  |  |
| Anxiety | 26.8 | 23.3 | .079 | 25.2 | 25.3 | .005 | 27.0 | 25.2 |
| Substance use disorder | 7.8 | 6.2 | .066 | 6.8 | 6.7 | .004 | 10.6 | 12.2 |
| Moderate/severe mental retardation | 1.4 | 0.4 | .113 | 0.2 | 0.2 | .001 | 7.4 | 8.1 |
| Neoplasms | 4.3 | 4.6 | .015 | 4.5 | 4.5 | .000 | 4.2 | 4.6 |
| Diabetes | 14.2 | 13.3 | .025 | 13.8 | 13.7 | .001 | 14.4 | 16.0 |
| Hyperlipidemia | 14.7 | 15.5 | .022 | 15.2 | 15.2 | .003 | 14.3 | 13.3 |
| White blood cell diseases | 1.0 | 0.9 | .013 | 0.9 | 0.9 | .003 | 1.2 | 0.8 |
| Anemia | 5.2 | 4.9 | .014 | 5.1 | 5.1 | .003 | 5.1 | 5.9 |
| Hypertension | 23.0 | 23.7 | .017 | 23.3 | 23.4 | .001 | 24.8 | 25.7 |
| Ischemic heart disease | 4.8 | 4.8 | .000 | 4.9 | 4.8 | .002 | 4.7 | 6.3 |
| Pulmonary circulation diseases | 0.3 | 0.2 | .007 | 0.2 | 0.3 | .005 | 0.3 | 0.09 |
| Cardiac dysrhythmias | 2.8 | 2.5 | .017 | 2.7 | 2.6 | .004 | 3.1 | 4.1 |
| Heart failure | 2.0 | 1.7 | .020 | 1.9 | 1.9 | .001 | 2.0 | 3.6 |
| Cerebrovascular disease | 3.2 | 2.5 | .042 | 2.8 | 2.7 | .005 | 3.7 | 4.6 |
| Acute bronchitis and bronchiolitis | 7.2 | 6.7 | .018 | 6.9 | 7.0 | .002 | 7.3 | 6.6 |
| Chronic bronchitis | 2.5 | 2.2 | .020 | 2.4 | 2.3 | .006 | 2.1 | 3.4 |
| Pneumonia | 1.9 | 1.7 | .016 | 1.8 | 1.8 | .001 | 2.5 | 2.6 |
| Emphysema | 0.6 | 0.6 | .007 | 0.6 | 0.6 | .002 | 0.6 | 0.7 |
| Asthma | 9.5 | 8.6 | .033 | 9.2 | 9.2 | .000 | 9.3 | 10.4 |
| Appendicitis | 0.1 | 0.1 | .001 | 0.1 | 0.1 | .004 | 0.3 | 0.1 |
| Noninfectious enteritis and colitis | 3.1 | 2.8 | .016 | 3.0 | 2.8 | .009 | 3.0 | 4.4 |
| Diverticula of intestine | 1.0 | 1.2 | .018 | 1.1 | 1.1 | .001 | 1.5 | 1.2 |
| Intestinal obstruction | 0.4 | 0.4 | .002 | 0.4 | 0.4 | .002 | 0.4 | 0.4 |
| Chronic liver disease and cirrhosis | 0.0 | 0.0 | .003 | 0.0 | 0.0 | .013 | 0.0 | 0.4 |
| Acute & chronic pancreatitis | 0.5 | 0.4 | .015 | 0.4 | 0.4 | .007 | 0.4 | 1.1 |
| Cholelithiasis | 0.9 | 1.0 | .005 | 0.9 | 1.0 | .002 | 1.2 | 1.2 |
| Acute kidney failure | 0.3 | 0.2 | .023 | 0.2 | 0.2 | .005 | 0.4 | 0.6 |
| Chronic kidney failure | 0.4 | 0.5 | .009 | 0.4 | 0.4 | .002 | 0.7 | 0.5 |
| Kidney infections | 0.5 | 0.4 | .014 | 0.5 | 0.5 | .001 | 0.8 | 0.6 |
| Cellulitis and abscess | 4.6 | 3.4 | .063 | 3.8 | 3.9 | .006 | 6.5 | 5.2 |
| Osteoarthrosis and allied disorders | 8.4 | 9.2 | .026 | 8.8 | 8.8 | .002 | 8.8 | 9.0 |
| Intervertebral disc disorder | 8.2 | 8.8 | .021 | 8.5 | 8.6 | .002 | 8.4 | 8.0 |
| Fractures, all sites | 3.8 | 3.1 | .039 | 3.4 | 3.4 | .003 | 4.4 | 5.4 |
| Fracture of neck of femur | 0.2 | 0.1 | .013 | 0.1 | 0.1 | .001 | 0.3 | 0.3 |
| Poisoning by psychotropic medication | 1.2 | 0.6 | .063 | 0.6 | 0.7 | .002 | 3.3 | 2.9 |
| Poisonings | 3.0 | 1.8 | .075 | 2.0 | 2.1 | .006 | 6.4 | 5.3 |
| Suicide and self-inflicted injury | 0.4 | 0.3 | .024 | 0.3 | 0.3 | .002 | 0.6 | 0.7 |
| Intracranial injury | 0.5 | 0.4 | .026 | 0.4 | 0.4 | .003 | 1.0 | 1.0 |
|  |  |  |  |  |  |  |  |  |
| Medication History, past 180 days |  |  |  |  |  |  |  |  |
| Psychotropic medication | 79.4 | 70.3 | .210 | 76.8 | 77.0 | .004 | 65.2 | 64.4 |
| Mood Stabilizer | 36.9 | 25.0 | .261 | 30.7 | 30.7 | .001 | 41.4 | 42.1 |
| ADHD medication | 7.5 | 5.1 | .097 | 6.1 | 6.1 | .001 | 9.8 | 9.7 |
| Anxiolytic/hypnotics | 67.1 | 60.7 | .132 | 65.5 | 65.8 | .006 | 54.6 | 53.4 |
| Other psychiatric medication | 1.2 | 0.9 | .029 | 1.0 | 1.1 | .002 | 1.4 | 1.0 |
| OB/GYN medication | 13.5 | 14.0 | .015 | 13.6 | 13.5 | .001 | 14.6 | 14.1 |
| Metabolic and related medication | 30.1 | 28.2 | .042 | 29.3 | 29.6 | .007 | 29.6 | 27.2 |
| Cardiovascular medication | 40.1 | 39.8 | .006 | 40.2 | 40.2 | .002 | 40.6 | 42.4 |
| Respiratory/allergy medication | 54.1 | 52.1 | .040 | 53.2 | 53.5 | .006 | 53.9 | 51.5 |
| Gastrointestinal medication | 44.1 | 42.5 | .033 | 43.6 | 43.6 | .001 | 43.1 | 42.4 |
| Neurologic/ musculoskeletal medication | 69.8 | 71.1 | .029 | 70.8 | 70.8 | .001 | 68.2 | 70.6 |
| Antibiotics | 52.0 | 50.7 | .026 | 51.2 | 51.4 | .004 | 52.7 | 49.7 |
| Diabetic medication | 13.4 | 12.5 | .024 | 13.1 | 13.1 | .000 | 12.8 | 14.1 |
| Hyperlipidemia medication | 23.2 | 22.0 | .029 | 22.9 | 23.2 | .007 | 21.2 | 20.0 |
|  |  |  |  |  |  |  |  |  |
| Acute services, past 180 days |  |  |  |  |  |  |  |  |
| Mental health emergency service | 9.5 | 6.1 | .126 | 7.9 | 6.6 | .053 | 14.2 | 11.4 |
| Psychotherapy | 34.1 | 30.9 | .070 | 33.3 | 32.1 | .025 | 34.1 | 31.6 |
| Psychosocial Service contacts | 5.2 | 3.4 | .090 | 4.2 | 3.8 | .019 | 7.4 | 8.3 |
| Number of OP visits for depression |  |  |  |  |  |  |  |  |
| 0-1 | 11.7 | 12.6 | .027 | 12.1 | 12.1 | .001 | 11.0 | 11.0 |
| 2 | 11.6 | 13.3 | .052 | 12.1 | 12.1 | .002 | 14.4 | 13.2 |
| 3 | 10.8 | 12.2 | .044 | 11.6 | 11.4 | .006 | 9.3 | 11.2 |
| 4 | 9.1 | 9.2 | .003 | 9.2 | 9.4 | .006 | 9.1 | 6.5 |
| 5-6 | 15.2 | 15.9 | .019 | 15.8 | 15.8 | .000 | 12.8 | 13.2 |
| 7-10 | 15.3 | 15.0 | .009 | 15.5 | 15.5 | .001 | 12.0 | 12.2 |
| 11-20 | 15.5 | 14.3 | .035 | 15.3 | 15.3 | .000 | 13.4 | 14.1 |
| 21+ | 10.8 | 7.6 | .110 | 8.4 | 8.4 | .000 | 18.1 | 18.5 |
| Number of MH hospital admissions |  |  |  |  |  |  |  |  |
| 0 | 94.7 | 97.1 | .121 | 96.7 | 96.7 | .000 | 87.1 | 87.9 |
| 1 | 4.3 | 2.5 | .100 | 2.9 | 2.9 | .001 | 9.2 | 8.5 |
| 2+ | 1.0 | 0.4 | .069 | 0.5 | 0.4 | .002 | 3.7 | 3.6 |
| Non-MH hospitalization | 8.5 | 7.2 | .047 | 7.9 | 7.5 | .016 | 10.0 | 10.4 |
| MH hospital admission | 5.3 | 2.9 | .121 | 3.3 | 3.3 | .000 | 12.9 | 12.1 |
| OP visits for depression (mean) | 10.1 | 8.0 | .148 | 8.5 | 8.4 | .011 | 15.6 | 16.1 |
| Non-MH ER visits (mean) | 0.9 | 0.7 | .078 | 0.8 | 0.7 | .013 | 1.2 | 2.8 |
| Non-MH outpatient visits (mean) | 16.3 | 14.4 | .104 | 14.8 | 14.8 | .000 | 21.6 | 22.8 |
| MH outpatient visits (mean) | 13.9 | 10.1 | .199 | 10.5 | 10.3 | .014 | 27.7 | 30.2 |
| Non-MH hospitalizations (mean) | 0.1 | 0.1 | .035 | 0.1 | 0.1 | .003 | 0.1 | 0.2 |
|  |  |  |  |  |  |  |  |  |
| State |  |  |  |  |  |  |  |  |
| AK | 0.3 | 0.3 | .007 | 0.2 | 0.3 | .005 | 0.6 | 0.3 |
| AL | 1.1 | 1.1 | .001 | 1.1 | 1.1 | .000 | 0.7 | 0.6 |
| AR | 0.9 | 0.9 | .002 | 1.0 | 0.9 | .004 | 0.3 | 0.7 |
| CA | 12.4 | 14.0 | .047 | 13.5 | 13.5 | .001 | 8.7 | 8.8 |
| CO | 0.3 | 0.3 | .001 | 0.3 | 0.3 | .001 | 0.1 | 0.2 |
| CT | 1.7 | 1.3 | .034 | 1.5 | 1.5 | .003 | 2.0 | 1.6 |
| FL | 3.1 | 3.0 | .008 | 3.2 | 3.2 | .002 | 2.0 | 1.7 |
| GA | 1.5 | 1.7 | .018 | 1.6 | 1.6 | .001 | 1.8 | 1.5 |
| HI | 0.3 | 0.4 | .015 | 0.3 | 0.3 | .003 | 0.3 | 0.4 |
| IA | 1.3 | 1.2 | .010 | 1.3 | 1.3 | .002 | 1.0 | 0.7 |
| ID | 0.7 | 0.6 | .021 | 0.6 | 0.6 | .001 | 1.2 | 1.3 |
| IL | 5.1 | 4.4 | .032 | 4.8 | 4.8 | .001 | 4.4 | 4.6 |
| IN | 3.3 | 2.5 | .051 | 2.9 | 2.8 | .001 | 3.7 | 3.9 |
| KS | 0.7 | 0.6 | .016 | 0.6 | 0.7 | .002 | 0.7 | 0.5 |
| KY | 2.5 | 3.6 | .063 | 2.7 | 2.7 | .000 | 5.2 | 5.0 |
| LA | 0.9 | 0.9 | .004 | 0.9 | 0.9 | .002 | 0.3 | 0.5 |
| MA | 3.0 | 2.2 | .048 | 2.6 | 2.5 | .001 | 3.6 | 3.7 |
| MD | 1.5 | 1.3 | .022 | 1.4 | 1.5 | .004 | 1.6 | 1.0 |
| ME | 1.1 | 1.3 | .022 | 1.1 | 1.1 | .005 | 1.2 | 1.7 |
| MI | 1.7 | 1.3 | .031 | 1.6 | 1.6 | .000 | 1.5 | 1.8 |
| MN | 2.8 | 3.2 | .021 | 2.8 | 2.8 | .002 | 3.6 | 3.5 |
| MO | 4.3 | 4.6 | .013 | 4.3 | 4.3 | .001 | 5.2 | 5.1 |
| MS | 0.9 | 1.0 | .012 | 1.0 | 1.0 | .002 | 0.6 | 0.8 |
| MT | 0.4 | 0.3 | .017 | 0.3 | 0.3 | .000 | 0.6 | 0.6 |
| NC | 4.1 | 3.4 | .040 | 3.8 | 3.6 | .008 | 4.2 | 6.6 |
| ND | 0.2 | 0.2 | .010 | 0.2 | 0.2 | .004 | 0.3 | 0.4 |
| NE | 0.9 | 0.5 | .043 | 0.6 | 0.6 | .002 | 1.7 | 1.2 |
| NH | 0.6 | 0.6 | .005 | 0.6 | 0.6 | .001 | 0.6 | 0.6 |
| NJ | 1.8 | 1.7 | .005 | 1.8 | 1.8 | .000 | 1.2 | 1.1 |
| NM | 0.7 | 0.8 | .008 | 0.8 | 0.7 | .007 | 0.3 | 0.9 |
| NY | 17.4 | 19.0 | .041 | 18.3 | 18.4 | .000 | 16.7 | 18.0 |
| OH | 0.0 | 0.0 | .013 | 0.0 | 0.0 | .000 | 0.0 | 0.0 |
| OK | 1.0 | 0.9 | .008 | 0.9 | 1.0 | .005 | 1.2 | 0.8 |
| PA | 1.9 | 1.7 | .013 | 1.9 | 1.9 | .001 | 1.0 | 0.9 |
| SC | 1.1 | 1.1 | .005 | 1.1 | 1.1 | .004 | 0.9 | 1.2 |
| SD | 0.2 | 0.2 | .001 | 0.2 | 0.2 | .001 | 0.1 | 0.2 |
| TN | 5.1 | 5.1 | .000 | 5.4 | 5.6 | .009 | 3.4 | 2.2 |
| TX | 4.0 | 2.6 | .077 | 3.1 | 3.1 | .002 | 5.7 | 4.3 |
| UT | 0.3 | 0.3 | .016 | 0.3 | 0.3 | .001 | 0.4 | 0.2 |
| VA | 1.6 | 1.3 | .024 | 1.5 | 1.5 | .003 | 1.2 | 0.8 |
| VT | 0.8 | 0.6 | .018 | 0.7 | 0.7 | .000 | 0.7 | 0.5 |
| WA | 1.6 | 1.6 | .007 | 1.7 | 1.7 | .001 | 0.8 | 0.7 |
| WI | 2.8 | 3.4 | .035 | 3.0 | 2.9 | .005 | 2.6 | 3.6 |
| WV | 2.2 | 3.5 | .078 | 2.4 | 2.4 | .001 | 5.5 | 5.0 |
| WY | 0.2 | 0.1 | .025 | 0.1 | 0.1 | .002 | 0.5 | 0.6 |
| ^a^After propensity score trimming and inverse probability of treatment weighting; Newer APM denotes the cohort initiating augmentation treatment with a newer antipsychotic; AD denotes the cohort initiating augmentation treatment with a second antidepressant medication; Std.Dif., standardized difference | | | | | | | | |

**eTable 4: Newer Antipsychotic Dose by Generic**

|  | **Index daily dose (n=25,172)** | | | | **Last fill daily dose* (n=15,153)** | | | |
| --- | --- | --- | --- | --- | --- | --- | --- | --- |
|  | **Actual mg** | | **CPZ** | | **Actual mg** | | **CPZ** | |
|  | **Mean** | **Median** | **Mean** | **Median** | **Mean** | **Median** | **Mean** | **Median** |
| Newer APMs combined (N=25,172) | n/a | n/a | 116.7 | 68.0 | n/a | n/a | 155.8 | 100.0 |
| Quetiapine (n=10,081) | 108.1 | 75.0 | 140.6 | 98.0 | 156.1 | 100.0 | 203.0 | 130.0 |
| Aripiprazole (n=4,342) | 7.0 | 5.0 | 94.0 | 67.0 | 9.0 | 5.0 | 119.9 | 67.0 |
| Olanzapine (n=3,935) | 7.3 | 5.0 | 145.2 | 100.0 | 8.7 | 6.0 | 174.9 | 120.0 |
| Risperidone (n=5,236) | 1.3 | 1.0 | 63.3 | 50.0 | 1.6 | 1.0 | 82.3 | 50.0 |
| CPZ, chlorpromazine equivalents; APM, antipsychotic medication  *Prior to censoring; limited to those with non-missing calculated daily doses; n=10,019 (39.8%) had no second fill after the index fill before censoring. | | | | | | | | |

**eTable 5: Dose-Response for All-Cause Mortality, N=39,582^a^**

|  | **Newer Antipsychotic  Augmentation** | | | **Antidepressant**  **Augmentation** | | |  |
| --- | --- | --- | --- | --- | --- | --- | --- |
| **Cause of Death** | **Deaths** | **Person Years** | **Incidence per 10,000 Person-Years** | **Deaths** | **Person Years** | **Incidence per 10,000 Person-Years** | **Adjusted Hazard Ratio (95% CI)** |
| **Newer Antipsychotic Dose Below Median (<68mg Chlorpromazine Equivalents)** | | | | | | | |
| All | 58 | 3,716 | 156.1 | 48 | 5,727 | 83.8 | 1.65  (1.11 to 2.44) |
| **Newer Antipsychotic Dose Above Median (≥68mg Chlorpromazine Equivalents)** | | | | | | | |
| All | 34 | 3,794 | 39.3 | 16 | 5,727 | 25.2 | 1.29  (0.85 to 1.95) |
| ^a^Pooled across all newer antipsychotics, based on index dose in chlorpromazine equivalents, after propensity score trimming and inverse probability of treatment weighting; 365 day maximum follow-up | | | | | | | |

**eTable 6: All-cause Mortality Stratified by Percentiles of the Propensity Score, N=44,301**

|  | **Newer Antipsychotic Augmentation** | | **Antidepressant Augmentation** | |  |
| --- | --- | --- | --- | --- | --- |
| **Propensity Score Percentile Range** | **Deaths** | **Person Years** | **Deaths** | **Person Years** | **Hazard Ratio (95% CI)** |
| 0 to 4.7^a^  (N=2,108) | 3 | 204 | 1 | 453 | 6.96  (0.72 to 66.9) |
| 4.7 to 25 (N=8,969) | 11 | 1,283 | 8 | 1,687 | 1.82  (0.73 to 4.52) |
| 25^th^ to 50  (N=11,074) | 24 | 1,966 | 18 | 1,731 | 1.17  (0.63 to 2.15) |
| 50 to 75  (N=11,097) | 31 | 2,313 | 11 | 1,434 | 1.73  (0.87 to 3.45) |
| 75 to 94.1^b^  (N=8,442) | 39 | 2,040 | 11 | 875 | 1.52  (0.78 to 2.96) |
| 94.1^b^ to 100  (N=2,611) | 12 | 836 | 8 | 172 | 0.30  (0.12 to 0.74) |
| ^a^Corresponding to the 2.5^th^ percentile of the propensity score distribution of the newer antipsychotic augmentation group; ^b^corresponding to the 97.5^th^ percentile of the propensity score distribution of the antidepressant augmentation group | | | | | |

**eTable 7: Mortality According to Underlying Cause of Death (Untrimmed Cohorts, Unadjusted), N=44,301^a^**

|  | **Newer Antipsychotic  Augmentation^b^** | | **Antidepressant Augmentation^c^** | |  |  |
| --- | --- | --- | --- | --- | --- | --- |
| **Cause of Death** | **Deaths** | **Incidence per 10,000 Person-Years** | **Deaths** | **Incidence per 10,000 Person-Years** | **Hazard Ratio (95% CI)** | **Rate Difference (per 10,000 years of follow-up)** |
| All^d^ | 120 | 138.9 | 57 | 89.7 | 1.54  (1.13 to 2.11) | 48.8  (11.7 to 99.6) |
| Natural | 78 | 90.3 | 36 | 56.7 | 1.59  (1.07 to 2.35) | 33.5  (4.0 to 76.5) |
| Non-Cancer | 77 | 89.1 | 34 | 53.5 | 1.66  (1.11 to 2.48) | 35.3  (5.9 to 79.2) |
| Un-Natural | 34 | 39.3 | 16 | 25.2 | 1.56  (0.86 to 2.83) | 14.1  (-3.5 to 46.1) |
| ^a^365 day maximum follow-up, as-treated; ^b^8,641 person years of follow-up; ^c^6,351 person years of follow-up; ^d^includes 13 deaths with unknown or missing cause of death | | | | | | |

**eTable 8: All-cause Mortality by Age Group, Sex, and Individual Newer Antipsychotic Medication (Untrimmed Cohorts, Unadjusted), N=44,301^a^**

|  | **Newer Antipsychotic Augmentation** | | **Antidepressant Augmentation** | |  |  |
| --- | --- | --- | --- | --- | --- | --- |
| **Subgroup** | **Deaths** | **Person Years** | **Deaths** | **Person Years** | **Hazard Ratio (95% CI)** | **Rate Difference (per 10,000 years of follow-up)** |
| *Age Group* |  |  |  |  |  |  |
| 25 to 54 | 73 | 6,867 | 34 | 4,920 | 1.53  (1.02 to 2.30) | 36.6  (1.4 to 89.8) |
| 55 to 64 | 47 | 1,774 | 23 | 1,431 | 1.65  (1.00 to 2.72) | 104.4  (0.0 to 276.5) |
|  |  |  |  |  |  |  |
| *Sex* |  |  |  |  |  |  |
| Female | 85 | 6,364 | 34 | 5,049 | 1.98  (1.33 to 2.94) | 66.0  (22.2 to 130.6) |
| Male | 35 | 2,277 | 23 | 1,302 | 0.88  (0.52 to 1.49) | -21.2  (-84.8 to 86.6) |
|  |  |  |  |  |  |  |
| *Generic Antipsychotic* |  |  |  |  |  |  |
| Quetiapine | 42 | 3,395 | 57 | 6,351 | 1.38  (0.92 to 2.05) | 34.1  (-7.2 to 94.2) |
| Risperidone | 27 | 1911 | 57 | 6,351 | 1.57  (0.99 to 2.48) | 51.2  (-0.9 to 132.8) |
| Aripiprazole | 16 | 1,443 | 57 | 6,351 | 1.24  (0.71 to 2.15) | 21.5  (-26.0 to 103.2) |
| Olanzapine | 29 | 1,376 | 57 | 6,351 | 2.33  (1.49 to 3.65) | 119.4  (44.0 to 237.8) |
| ^a^365 day maximum follow-up, as-treated | | | | | | |

**eTable 9: Sensitivity Analyses (Untrimmed Cohorts, Unadjusted), N=44,301**

|  | **Newer Antipsychotic Augmentation** | | **Antidepressant Augmentation** | |  |  |
| --- | --- | --- | --- | --- | --- | --- |
|  | **Deaths** | **Person Years** | **Deaths** | **Person Years** | **Hazard Ratio (95% CI)** | **Rate Difference (per 10,000 years of follow-up)** |
| *Follow-up Specification* |  |  |  |  |  |  |
| 365-day follow-up As-Treated | 120 | 8,641 | 57 | 6,351 | 1.54  (1.13 to 2.11) | 48.5 (11.7 to 99.6) |
| 365-day follow-up ITT | 307 | 22,270 | 156 | 16,945 | 1.50 (1.24 to 1.82) | 46.0 (22.1 to 75.5) |
| 180-day follow-up As-Treated | 88 | 6,617 | 42 | 4,932 | 1.56  (1.08 to 2.25) | 47.7 (6.8 to 106.4) |
| 180-day follow-up ITT | 164 | 11,692 | 74 | 8,893 | 1.69 (1.28 to 2.22) | 57.4 (23.3 to 101.5) |
| All days in study period As-Treated | 157 | 11,325 | 81 | 8,064 | 1.37  (1.05 to 1.79) | 37.2 (5.0 to 79.4) |
| Censoring for discontinuation of baseline antidepressant | 83 | 6,670 | 36 | 4,952 | 1.69  (1.14 to 2.50) | 50.2 (10.2 to 109.0) |
|  |  |  |  |  |  |  |
| *Exclusion criteria* |  |  |  |  |  |  |
| Excluding mood stabilizer use during baseline period | 72 | 7,372 | 31 | 4,675 | 1.99  (1.31 to 3.04) | 65.6 (20.6 to 135.3) |
|  |  |  |  |  |  |  |
| *Index Year^a^* |  |  |  |  |  |  |
| *2007-2010* | 42 | 2,845 | 13 | 1,876 | 2.14  (1.15 to 3.98) | 79.0  (10.4 to 206.4) |
| ^a^365 day maximum follow-up, as-treated | | | | | | |

**eTable 10: Mortality According to Underlying Cause of Death (Untrimmed Cohorts, Adjusted for Age, Sex, Race/Ethnicity and Index Year), N=44,301^a^**

|  | **Newer Antipsychotic  Augmentation^b^** | | **Antidepressant Augmentation^c^** | |  |  |
| --- | --- | --- | --- | --- | --- | --- |
| **Cause of Death** | **Deaths** | **Incidence per 10,000 Person-Years** | **Deaths** | **Incidence per 10,000 Person-Years** | **Adjusted Hazard Ratio (95% CI)** | **Rate Difference (per 10,000 years of follow-up)** |
| All^d^ | 120 | 138.9 | 57 | 89.7 | 1.54  (1.12 to 2.12) | 45.3 (10.1 to 93.9) |
| Natural | 78 | 90.3 | 36 | 56.7 | 1.61  (1.08 to 2.40) | 32.0 (4.2 to 73.4) |
| Non-Cancer | 77 | 89.1 | 34 | 53.5 | 1.69  (1.12 to 2.53) | 33.7 (5.9 to 74.8) |
| Un-Natural | 34 | 39.3 | 16 | 25.2 | 1.47  (0.81 to 2.68) | 11.5 (-4.6 to 41.0) |
| ^a^365 day maximum follow-up, as-treated; ^b^8,641 person years of follow-up; ^c^6,351 person years of follow-up; ^d^includes 13 deaths with unknown or missing cause of death | | | | | | |
