# Supplementary material for: Mortality risk of antipsychotic augmentation for adult depression
Source: PLoS One. 2020 Sep 30;15(9):e0239206. doi: 10.1371/journal.pone.0239206 (PMC7526884; doi:10.1371/journal.pone.0239206)
Supplement: S2 Appendix — (PDF) [file pone.0239206.s002.pdf]

Safety of Second Generation Antipsychotics for Adult Depression

Major depressive disorder (MDD) affects approximately 15.7 million US adults and causes substantial emotional, physical, and economic burden.[1-3] Inadequate response to pharmacological treatment is common and multiple sequential treatment steps are often required to achieve remission; 60-70% of patients fail to achieve remission from the first antidepressant trial.[4] Clinical strategies to treat incomplete response include switching to another antidepressant and various augmentations.[5-7] Augmentation with second-generation antipsychotics (SGAs) is arguably the best supported and the only FDA approved, treatment alternative for MDD patients with ≥2 failed trials of antidepressant monotherapy.[6,8] While the efficacy of SGA augmentation for depression has been established in numerous randomized controlled trials (RCTs),[9-23] evidence concerning its safety, particularly for risks for rare, but serious adverse events, including premature mortality, remains underdeveloped due to the restricted inclusion criteria, short duration, and limited sample sizes of these RCTs.[8,24] This is a major public health concern, as (1) SGAs have been associated with increased risks for rare but serious adverse effects in other indications,[25-30] particularly a >50% increased mortality risk in older adults with dementia;[30] (2) the benefits of SGA augmentation for MDD are modest with a number needed to treat for remission of about 9[8,24] and little evidence for improvement in overall well-being;[24] (3) use of SGA augmentation in patients with depression increased more than 2.5 fold from 1999-2010, and it was prescribed in ~2 million visits to office-based physicians per year in 2009-10.[31] The uncertain safety profile and modest benefits of SGA augmentation for depression raise concerns about potential overuse, particularly compared to non-pharmacological treatment options that are subject to significant access barriers.[32,33]

Using the most recent 10 available years of near national Medicaid data, merged with National Death Index and Area Resource File data, we will estimate the real world safety of SGAs in ~80,000 non-elderly adults with depression and incomplete response to antidepressant monotherapy. Individuals diagnosed with psychotic depression or other established indications for antipsychotic therapy will be excluded. The inferential analyses proposed in Aims 2 and 3, including the feasibility of an instrumental variable (IV) analysis, will be informed by a rigorous examination of the epidemiology of augmentation strategies in depression (**Aim 1**). All inferential analyses will employ an active comparator inception cohort design. We will initially compare the incidence of serious adverse events (all-cause mortality, sudden cardiac death, acute myocardial infarction (AMI), stroke, type 2 diabetes, venous thromboembolism (VTE), pneumonia) between patients initiating new episodes of SGA augmentation and those initiating antidepressant augmentation (**Aim 2a**). Next, we will examine the safety of individual SGA augmentation strategies (**Aim 2b**). Lastly, to facilitate personalized treatment, we will explore potential treatment effect heterogeneity by age and severity of cardiovascular disease (**Aim 3**). Bias, particularly confounding by indication, will be minimized by design (inception cohort, relevant active comparators, careful restriction of the study population) and through statistical strategies to minimize confounding and assess the robustness of results (propensity scores, sensitivity analyses). If a suitable instrument based on treatment preference at the geographic or prescriber level can be identified, we will implement IV estimation to further address potential bias from residual confounding. The specific aims are to:

Aim 1: Characterize the epidemiology of alternative augmentation strategies for adult non-psychotic depression.

a) Determine the annual prevalence and predictors of SGA augmentation.

b) Compare the characteristics of SGA and antidepressant augmentation treatment episodes.

Aim 2: Estimate class and drug-level risks for all-cause mortality and serious adverse events (sudden cardiac death, AMI, stroke, type 2 diabetes, pneumonia, and VTE) of SGA augmentation in adult non-psychotic depression.

a) Compare risks of serious adverse events associated with SGA augmentation and antidepressant augmentation.

b) Within SGA augmentation treatment episodes, compare risks of serious adverse events among commonly prescribed SGAs.

Aim 3: Explore potential treatment effect heterogeneity and dose/duration-effects in the safety profiles of alternative augmentation strategies. For class and drug-level comparisons, examine potential heterogeneity in the comparative safety of SGA augmentation in subgroups defined by age and baseline cardiovascular disease severity and examine safety risks by duration of follow-up and drug dose.

The results of our study will be a first step to help inform clinical, regulatory, and health care policy efforts to improve the management of treatment resistant depression. Our study will directly inform prospective safety studies, such as large simple trials or registry studies, by strengthening or refuting important safety hypotheses and thus facilitate a more conclusive examination of benefits and harms of SGA augmentation in depression.

2.A. SIGNIFICANCE

2.A.1. Depression: Prevalence and Impact. According to the World Health Organization, depression robs North Americans of more years of health than any other disease.[34] During the course of 1 year, 7% of US adults experience an episode of major depression, imposing a heavy burden on the affected individuals, their families, and their communities.[3] Depression severely compromises quality of life, marital stability, and occupational productivity, while increasing risk of suicide and cardiovascular (CV) disease.[35-41]

2.A.2. Treatment of Depression. Antidepressants (ADs) are used by roughly one in ten Americans each year.[42] Yet only about one-half of depressed patients respond to an initial AD medication.[4,43] For those who do not, switching to another AD is recommended, followed by a variety of augmentation medications.[5] Although treatment with two concurrent ADs is the most common augmentation strategy for "treatment-resistant" depression,[31] addition of a second-generation antipsychotic (SGA) remains the best-studied, and only FDA approved, adjuvant treatment for depression.[6-8,24]

2.A.2.1. SGA Augmentation for Depression. Over the last 15 years, there has been growing evidence for efficacy of SGAs as adjunctive treatments for depression.[9-23] Since 2007, the FDA has approved olanzapine with fluoxetine, aripiprazole, and quetiapine as adjuncts to ADs for adults with MDD and inadequate response to ADs alone. Effect sizes are modest for remission (OR 2.00, 1.58-2.72, NNT=9) and response (OR 1.69, 1.46-1.95, NNT=9) with no significant efficacy differences between individual SGAs.[8] No randomized controlled trials (RCTs) have directly compared individual SGAs as adjunctive treatments for MDD.

2.A.2.2. Utilization of SGA Augmentation. The role of SGA augmentation in clinical practice is not well characterized, though it is a highly marketed treatment strategy.[44] The only nationally representative utilization estimates are our estimates (see 2.C.2.1) that SGAs were prescribed in 12.5% of office-based physician visits for non-psychotic depression in 2009-10, a >2.5 fold increase since 1999-2000.[31] Notably, use rates exceeding 7% were observed well before the first FDA approval of SGA augmentation for depression in 2007. Such off-label use following publication of successful trial results but prior to FDA approval is not unusual.[21]

2.A.3. Evidence Gaps for SGA Augmentation. Serious adverse effects of SGAs in other clinical populations underscore the need of increasing our understanding of the safety of SGA augmentation in depression.[25-30] The most serious of these adverse effects is a >50% increase in mortality for SGAs above placebo in older adults with dementia,[30] which has prompted a FDA boxed warning.[45] Due to limited sample sizes of the RCTs, the safety profile of SGA augmentation remains poorly characterized. For a moderately rare adverse event such as all-cause mortality, which in this population occurs at a rate of ~1.6%/year (2.C.6), even pooled data from all existing RCTs[8] provide insufficient power to detect a 4-fold increase in risk. Observational studies of the safety of individual SGAs in older adults found similar patterns of risk across age categories ≥age 65 and for patients with and without dementia.[46,47] These findings heighten concerns regarding the generalizability of the increased mortality risk in older adults with dementia to less frail non-elderly MDD patients. Specific depression-associated SGA safety concerns are raised by depression linked immune response activation,[48,49,50] and increased risks of CV disease.[35,41] Risks may also be increased in SGA-naïve patients,[27,51] a group that is likely overrepresented in patients with non-psychotic depression. Differential mortality risks among individual SGAs observed in older adults[46,47] establish the need to examine their individual safety in depression.

2.A.4. Conceptual Model of SGA-Related Mortality in Depression. Multiple biological pathways have been proposed to explain SGA-associated mortality in various clinical populations.[27,52-54] The large number of potential mechanisms prevents direct inferences regarding the generalizability of this risk to depression. To inform our safety study, we have developed a working model for SGA-associated mortality risk in depression. The model includes pathways resulting from SGA effects on (1) depression, [8,24,35,36,38,40] (2) QT prolongation,[53,55,56] (3) sedation/extra pyramidal symptoms/immobility,[52,54,57] and (4) weight gain/dyslipidemia.[25,27,28] Because depression is a risk factor for CV disease[35,41] and death[35,38,40] (including suicide),[36] improved control of depression through SGA augmentation might partially offset SGA-related adverse effects. An exploration of cause of death data (2.C.7.2) will strengthen our understanding of mechanisms of SGA-associated mortality in depression. The rise in SGA augmentation for depression brings urgency to developing a greater understanding of SGA safety in this clinical context. We have learned from other SGA indications, particularly dementia in older adults, that without determined efforts to examine risks for rare, but serious, adverse events, substantial harm can go undetected for many years.[58] It is vital to identify such risks early to minimize the public health impact.[59]

Figure 1: Working Conceptual Model

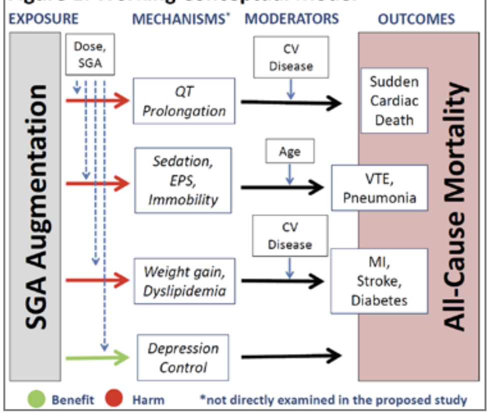

2.B. INNOVATION The proposed study breaks new ground by extending safety research on SGAs, which has been largely confined to older adults, especially those with Alzheimer's disease, and young patients with schizophrenia, to the large and growing group of patients who receive SGAs as an adjuvant treatment for depression. It addresses the challenging issue of how best to use large observational datasets to examine the risk of rare, but severe, adverse effects that are unlikely to be detected by RCTs. Using modern pharmaco-epidemiologic methods, it innovatively applies insights from mental health services research to emerging patterns of psychotropic drug utilization. The study leverages existing data resources at the Rutgers CERTs to enable novel class- and drug-level analyses that will inform development and design of future safety studies.

2.C. APPROACH

2.C.1. Overall Strategy. Augmentation with a SGA is an increasingly widely used treatment for depressed patients who do not remit with AD monotherapy. Yet little is known about the safety of SGAs as adjuvant treatment for adult depression. The proposed safety study (**Figure 2**) addresses this critical gap by comparing rare but serious adverse outcomes between initiators of SGA augmentation and combination treatment with two ADs in 10 years of national Medicaid data. We focus exclusively on safety as effectiveness is better examined in RCTs. Key decisions in the conceptualization of the proposed study are summarized below.

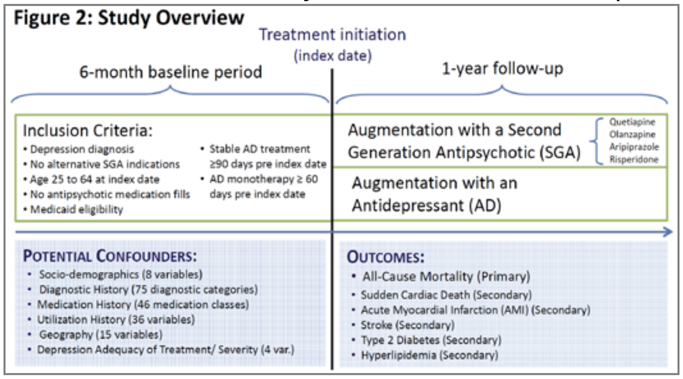

Figure 2: Study Overview

2.C.1.1. Study Population. The study focuses on depressed adults who have moved beyond AD monotherapy. ***Rationale:*** (1) These groups represent critical populations for which accurate safety data are much-needed, as the benefit effect sizes are modest and effective non-pharmacological treatment options exist.[5,8,32] (2) By restriction to those who do not respond to monotherapy, we focus on a relatively homogeneous population and thus reduce confounding by indication.[60]

2.C.1.2. Study Period. The study will utilize data from CYs 2001-2010. **Rationale:** Significant rates of SGA augmentation for depression have been observed as early as 2001 when initial trial data became available.[21] To account for potential changes in treatment selection/preferences after FDA approval in 2007, we will replicate analyses limited to post FDA approval (2007-10) and include the most recently available data.[61]

2.C.1.3. Control Group. Our study will utilize active comparison groups. We will compare SGA and AD augmentation (**Aim 2a**) and, among initiators of SGA augmentation, compare each individual SGA to quetiapine (reference drug, **Aim 2b**). ***Rationale:*** (1) Direct comparisons between alternative active treatments minimize confounding by indication.[62] We will limit comparisons to AD augmentation rather than monotherapy as the latter tends to occur at an earlier stage of depression treatment.[5,6,43] (2) Quetiapine was chosen as the referent SGA because it shows the lowest mortality risk in elderly patients with and without dementia.[46,47,63]

2.C.1.4. Study Design. We propose a retrospective cohort study of new initiators of alternative augmentation strategies for depression. ***Rationale:*** (1) A sufficiently large RCT or prospective cohort study is premature without strong evidence of safety risks from high-quality observational research. The present study will help support or refute the need for such research. (2) A new user design minimizes selection bias.[64,65]

2.C.1.5. Outcomes and Effect Modifiers. The *primary outcome* of this safety study is all-cause mortality. *Secondary outcomes* are sudden cardiac death, AMI, stroke, type 2 diabetes, pneumonia, and VTE. Effect modifiers include age and severity of CV disease. ***Rationale:*** (1) SGAs have been associated with each of these outcomes in other populations,[25-30,52,66-69] but not rigorously examined in non-elderly depressed patients. (2) All outcomes are validated or well established in claims or the National Death Index (**Table 2**).
(3) Examination of heterogeneity in adverse effects will inform personalized risk-benefit assessments. Age was selected because the most robust premature mortality findings have been limited to older adults.[30] CV disease severity has been previously identified as a moderator of SGA-associated sudden cardiac death.[29]

2.C.1.6. Analytic Methods. We will use multivariate Cox regression,[70] adjusted for an extensive set of patient characteristics (**Table 3**) using propensity score (PS) techniques to minimize potential confounding.[71,72] Residual confounding will be assessed in quantitative sensitivity analysis and instrumental variable estimation. ***Rationale:*** (1) We chose Cox models to allow examination of the time-varying nature of treatment effects,[70] and to avoid underestimation of adverse event risks.[73] (2) PSs offer significant advantages over multivariate regression in studies of infrequent outcomes.[74] Because the PS models the relation of covariates with drug exposure and not directly with rare study outcome, PS modeling avoids the risk of over-fitting and facilitates adjustment for a large set of potential confounders.[75] (3) To address potential bias from unmeasured confounders, we will conduct quantitative sensitivity analysis[76] and explore instrumental variable analysis.[77]

2.C.1.7. Study team. Led by Dr. Gerhard, the team has an established collaborative track record, extensive experience with safety studies in large databases, and spans a broad range of expertise necessary for the successful completion of the present study. Drs. Gerhard, Crystal, Olfson, and Correll are recognized experts in pharmacoepidemiology, mental health services research, psychiatry, and clinical psychopharmacology.

2.C.2. Preliminary Studies

2.C.2.1. National Trends in SGA Augmentation. In a recent analysis of the National Ambulatory Medical Care Survey, we found a nearly threefold increase in SGA treatment of depression in office-based practice from 1999 to 2010 (AOR: 2.78, 95% CI: 1.84-4.20).[31] In 2009-10, SGAs were used in 12.5% of ~37 million visits for depression in which ADs were prescribed. Notably, by 2001-02 SGA use exceeded 7%.

2.C.2.2. Preliminary Analyses in Medicaid Analytic eXtract (MAX) Data. To assess the feasibility of the proposed study, we used 5 years (2001-05) of national MAX data to estimate sample sizes. We identified 81,944 augmentation episodes, of which 67,067 were for AD augmentation and 14,877 for SGA augmentation. To distinguish AD augmentation from AD switching, we examined treatment continuation rates at 60, 90, and 120 days after index date (**Table 1**). We observed significantly lower on-treatment rates for the AD cohort (specification I) than the SGA cohort. We thus required a refill of the baseline AD on the index date (specification II) to reduce the likelihood of including AD switchers. Using this approach, on-treatment rates increased by ~40% for the AD augmentation cohort and on-treatment rates were virtually identical to the SGA augmentation cohort.

Table 1. Percent Treatment Continuity over Follow-up.

	Antidepressant Augmentation		SGA Augmentation
	Specification I n=67,067	Specification II n=15,381	n= 14,877
**Day 60**	51.3%	69.0%	70.6%
**Day 90**	43.7%	60.3%	62.1%
**Day 120**	38.0%	53.4%	55.4%

To inform the feasibility of an instrumental variable (IV) analysis, we examined county level rates of SGA augmentation.[78] Among individual counties with ≥10 cohort patients, the proportion with SGA vs. AD augmentation varied from 20% to 93% (IQR=46%-62%). While part of the between county variation is likely explained by differences in case-mix, the magnitude of observed variation suggests that factors exogenous to individual patients such as prescriber preferences make substantial contributions. We will explore the utility of using this exogenous variation as a preference-based instrument in IV estimation (2.C.7.2.2).[78-80]

2.C.3. Setting and Sources of Data. All data necessary for the completion of the proposed project either exist at Rutgers or will be established by the start date through scheduled data acquisitions for the CERTs.

2.C.3.1. Medicaid Analytic eXtract (MAX) Data, 2001-10. MAX files include a Personal Summary File with enrollment information and 4 claims files (Inpatient, Other Therapy, Long Term Care, and Prescription Drug). Pharmacy claims provide National Drug Codes, fill dates, quantity, and days supplied. To rule out data validity concerns for patients receiving benefits through comprehensive managed care, we will evaluate the completeness and quality of each file type by state and calendar year using established reference metrics.[81,82]

2.C.3.2. National Death Index (NDI) and Area Resource File (ARF). Date and cause of death will be identified by linkage to the National Death Index (NDI), maintained by the National Center for Health Statistics (NCHS) and regarded as the most authoritative source of death information for the US population, including over 99% of all deaths in the US.[83-86] The ARF provides county-level information on community characteristics and resources including health care delivery systems and factors that may impact health status and care.[87]

2.C.4. Study Population. The proposed study will compare all-cause mortality and select severe adverse outcomes between 2 adult (25-64 years) cohorts with depression who, after AD monotherapy, either: 1) add a second AD or 2) add a SGA. The study population will be selected from Medicaid enrollees in 45 US states (~95% of all US Medicaid enrollees) from 2001-2010. Initiation of augmentation defines the index date and the beginning of follow-up. Eligibility criteria and covariates are assessed during the 180 days immediately preceding the index date. Longer pre-index periods will be explored (2.C.7.1). Patients will have uninterrupted Medicaid coverage during the pre-index period, ≥1 primary in- or outpatient diagnosis of depression in the first 90 days of the pre-index period (ICD-9-CM 296.2, 296.3, 300.4, 311; similar depression claims algorithms have demonstrated PPVs >80% compared to patient self-reports or medical record diagnoses),[88-90] and no break of >14 days in medication supply with a single AD medication during the 90 days directly preceding the index date. For patients initiating a second AD, we will also require that the initial AD is refilled on the same day to maximize the likelihood that the clinical intent was to combine treatment of 2 ADs (2.C.2.2).

Patients with alternate indications for SGAs (psychotic depression, schizophrenia, bipolar disorder, autism-spectrum disorders, or dementia) will be excluded, maximizing the likelihood that SGA treatment was initiated for depression. Patients will also be excluded for any antipsychotic medication use during the baseline period and for use of a second AD during the 60 days immediately preceding the index date, as this likely indicates a greater degree of treatment resistance.[91-93] Patients who initiate a second AD and a SGA on the index date or

who are diagnosed with a life threatening disorder[29] will also be excluded. For secondary outcome analyses, patients will be excluded if they have diagnosis or treatment for the respective outcome in the pre-index period.

2.C.5. Measures.

2.C.5.1. Exposure and Follow-up Time. SGAs for **Aim 2b** include risperidone, quetiapine, aripiprazole, and olanzapine. All other SGAs combined make up <5% of SGA augmentation in MDD[31] and thus do not facilitate meaningful analyses. ADs include SSRIs, SNRIs, TCAs, bupropion, and mirtazapine.[5] In sensitivity analysis, agents that may be used for indications other than augmentation for depression will be excluded to assure that their potentially inappropriate inclusion does not bias the results (TCAs; mirtazapine and quetiapine at low doses). ADs and SGAs will be identified from MAX pharmacy files that include generic and brand names, dose, dispensing date and quantity, and days of supply dispensed. Computerized pharmacy records are highly accurate and not subject to recall bias.[94-97] Each day of follow-up will be classified according to probable use of each AD and SGA agent. For each new augmentation episode, we will create a diary of coverage by linking consecutive fills. Patients will be classified as discontinuing when either drug is not refilled for >14 days.[98] A 14 day buffer is used to account for late refills and stockpiling. Follow-up will be capped at 365 days because by this point a majority of patients have discontinued index treatment (2.C.2.2). Follow-up of >365 days will be examined in exploratory analyses and interpreted with appropriate caution. SGA dose will be classified in chlorpromazine equivalents[99] to allow comparisons across agents and examine dose-response.

2.C.5.2. Outcomes. Definitions and measurement characteristics of study outcomes are shown in **Table 2**.

Table 2 Primary and Secondary Outcomes

Primary Outcome	Definition and Measurement Characteristics
All-Cause Mortality	Occurrence and date of death determined by the National Death Index (NDI). Specificity 100%, sensitivity 98-100%[83,85]
**Secondary Outcomes**	**Definition and Measurement Characteristics**
Sudden Cardiac Death	Validated algorithm based on cause of death (NDI)[86] and Medicaid claims. Positive predictive value (PPV) 87%[100]
Acute Myocardial Infarction	First or second listed hospital discharge diagnosis of ICD-9 code 410. (PPV of 89%-97%)[101-105]
Stroke	First listed hospital discharge diagnosis of ICD-9 codes 430, 431, 433.x1, 434.x1, or 436 (PPV 92%-100%)[106-108]
Type 2 Diabetes	Physician claim with ICD-9 codes of 295.00-295.93, 357.2, 362.0-362.02, or 366.41. (Specificity 93%-97%)[109,110]
Pneumonia	First listed hospital discharge diagnosis of ICD-9 codes 480-486, and 507.[69]
Venous Thromboembolism	ICD-9 codes 415.x (pulmonary embolism), or 451.x (deep vein thrombosis) and >1 anticoagulant claim. (PPV 93%)[111]

2.C.5.3. Confounding. Confounding by indication is a key challenge in all pharmacoepidemiologic studies as physicians make treatment choices in light of available clinical information.[112,113] We will minimize confounding through restriction (2.C.1.1) and active comparators (2.C.1.3). We will also control for a broad range of clinically relevant factors from claims histories and the ARF using propensity score techniques (2.C.1.6). This will involve >180 variables encompassing socio-demographics; diagnosis, medication, and utilization history; and geography as well as a set of variables that specifically characterize the adequacy of pharmacological treatment[114] and the severity of depression during the pre-index period (**Table 3**).

Table 3 Potential Confounding Variables (assessed during the 180-day pre-index period)

Confounder Category		Source
Socio-demographics	Age, sex, race/ethnicity, Medicaid enrollment category (8 variables)	MAX Personal Summary File
Diagnostic and Medication History (75 diagnostic categories; 45 medication classes)	Psychiatric (18 dx categories; 15 rx classes), OB/GYN (13 dx categories; 3 rx classes), Metabolic (14 dx categories; 4 rx classes), CV (3 dx categories; 4 rx classes), Respiratory/Allergy (8 dx categories; 5 rx classes), GI (3 dx categories; 7 rx classes), Neurologic/musculoskeletal (8 dx categories; 7 rx classes), Other (8 dx categories)	MAX Inpatient and Other Claims Files;<br>MAX Prescription Drug File
Utilization History (36 variables)	Number and/or duration of outpatient visits, inpatient stays, ED visits, and prescription fills during 0-7 days, 8-30 days, and 31-180 days prior to the index date	MAX Claims and Prescription Drug Files
Geography (15 variables)	State and county level markers of supply of health facilities and health professionals, health status, economic activity, socioeconomic and environmental characteristics.	Area Resource File
Depression: Adequacy of Treatment and Markers of Severity(4 variables)	Adequacy of depression treatment (scored 0-5) as calculated by an algorithm based on antidepressant agent, dose, duration, and adherence.[114] Number of outpatient visits for depression; ED visits for depression; inpatient stays for depression.	MAX Claims and Prescription Drug Files

2.C.5.4. Effect Modification. The examination of potential treatment effect heterogeneity will consider age group (25-34, 35-44, 45-54, 55-64), and severity of cardiovascular disease (none, mild, moderate, severe) based on a claims-based cardiovascular risk score.[53]

2.C.6. Sample Size and Power. **Table 4** shows detectable hazard ratios for mortality at 80% power and a 5% alpha for class- (**Aim 2a**) and drug level (**Aim 2b**) comparisons. Expected cohort sizes are based on 2001-05 MAX data (2.C.2.2) and were projected conservatively to the proposed study periods (2001-10 and 2007-10). Calculations assume a mean time to discontinuation of 230 days and a background mortality rate of 1.6%/year (also estimated from MAX, 2001-05). Our study is powered to detect a hazard ratio for all-cause mortality of 1.21 (1.31 for 2007-10), markedly smaller effect sizes than the >50% risk increase demonstrated for SGAs in older adults with dementia. This substantial sample size facilitates examination of risks for individual SGAs, the period after FDA SGA augmentation approval, and clinically important subgroups.

2.C.7. Analytic Strategy

2.C.7.1. Epidemiology of SGA Augmentation (Aim 1). We will first identify all periods of SGA augmentation among patients with depression without alternative indications for SGA treatment. Building on prior work,[31] we will examine prevalence and predictors of SGA augmentation for each calendar year to evaluate trends and patterns of prevalent SGA use in non-psychotic depression. We will then identify all incident episodes of SGA and AD augmentation (2.C.5.1). For each episode, we will examine treatment and diagnosis patterns in the preceding 180 days. We will also examine longer look-back windows (12, 18, and 24 months) to assess enrollment patterns outside of the 180 day window and to determine whether longer look-back periods allow more extensive characterization of treatment and diagnostic history, particularly regarding the severity and duration of depression. Number and duration of trials of AD monotherapy and alternative augmentation agents (lithium, buspirone, and T_3) will be determined as proxies for treatment resistance.[91-93] We will then compare the cohorts (across classes and across individual SGAs) by baseline characteristics to assess the magnitude of potential confounding and examine alternate cohort definitions with longer look-back windows that may further reduce confounding. Group differences will be assessed using standardized differences to allow meaningful examination of the magnitude of relative differences independent of sample size.[115] We will also characterize treatment duration post-index treatment, and dosing (including SGA initiation doses and titration patterns over follow-up).[116]

Table 4: Sample Size and Detectable Hazard Ratios

Treatment	Number of Episodes		Detectable Hazard Ratio (All-cause Mortality)	
	**2001-2010**	**2007-2010**	**2001-2010**	**2007-2010**
**Aim 2a (Class-Level Comparison)**				
AD Augmentation	40,298	19,907	*reference*	*reference*
SGA Augmentation	38,891	19,211	1.21	1.31
**Aim 2b (Comparison by Individual SGA)**				
Quetiapine	11,303	5,584	*reference*	*reference*
Olanzapine	10,523	5,198	1.42	1.63
Risperidone	9,678	4,781	1.43	1.64
Aripiprazole	4,646	2,295	1.53	1.79

Preliminary data (2.C.2.2) suggest that treatment preference at the prescriber[117] or geographic[118] level may serve as an instrumental variable (IV). More detailed analyses under **Aim 1** will explore this geographic and provider-level variation in SGA augmentation. Similar to prior work by our group,[79] these analyses will fit mixed-effects logistic regression models to estimate county- and provider-specific prescribing rates adjusted for case-mix and regional characteristics from the ARF to identify a suitably strong instrument (2.C.7.2.2).

Lastly, we will determine the size and compare the characteristics of our study cohorts to similar Medicaid cohorts with relaxed eligibility requirements to assess whether such restriction affects generalizability.

2.C.7.2. Comparative Safety (Aim 2). We will plot Kaplan-Meier curves for each augmentation strategy and outcome (2.C.5.2) to depict crude event rates by duration of use.[119] We will then use Cox proportional hazards regression to model the effect of alternative augmentation strategies on these adverse health outcomes. To examine the safety of SGA augmentation (**Aim 2a**), we will compare the risk of adverse outcomes between new treatment episodes of SGA initiators and initiators of AD augmentation. To examine the safety of individual SGAs used for augmentation (**Aim 2b**), we will compare new treatment episodes of quetiapine and the other individual SGAs. Follow-up starts on the day of augmentation initiation and end on the day patients develop a study outcome, discontinue augmentation therapy (plus 30 days to avoid under-ascertainment of outcome events), start a different AD or SGA, reach day 365, or leave the study population, whichever comes first. In sensitivity analyses, we will (1) restrict the study period to treatment initiations on or after 01/01/2007, (2) extend the maximum follow-up beyond day 365, and (3) model cumulative relative risk for outcomes during 180, 365, and 730 days of follow-up. Initially, we will fit unadjusted models and models that control for sex, age, and race/ethnicity. Because diagnosis of diabetes requires testing, we will perform sensitivity analyses that account for laboratory testing and office visits with metabolic screening to minimize detection bias.[120]

To control for confounding related to initiation (**Aim 2a**) or choice (**Aim 2b**) of SGA augmentation, we will use propensity score (PS) techniques.[72] For each comparison, PSs will be calculated using non-parsimonious logistic regression based on a large set of potential confounds (**Table 3**). We will optimize PS performance by examining standardized differences in covariate rates between treatments before and after stratification.[115] To assure equipoise, we will exclude patients in non-overlapping tails of the PS distributions.[121] If significant changes in prescribing patterns over the study period become apparent, we will explore calendar time-specific PSs.[122] Separate Cox models that control for sex, age, race/ethnicity, and the comparison-specific PS decile will be fit for each comparison. Lastly, if we observe an increased all-cause mortality risk, we will examine the distribution of causes of death to inform future mechanistic work on SGA associated mortality in depression.

2.C.7.2.1. Quantitative Sensitivity Analyses for Unmeasured Confounders. Because the observed relative risk is a closed-form function of the balance of confounders among exposure groups, the strength of the confounder, and the prevalence of the confounder, it is possible to estimate the extent of unmeasured and thus unadjusted confounding (e.g., by BMI or family history) necessary to fully explain the observed findings.[76]

2.C.7.2.2. Instrumental Variable (IV) Analysis. To further address residual confounding, we will implement IV estimation,[123,124] which can provide unbiased estimates of causal effects in non-randomized studies even if confounders remain unmeasured[77] by mimicking random assignment of patients into groups of different likelihood for treatment.[125,126] This method relies on the existence of an instrument that is closely related to the actual treatment choice but is unrelated to patient characteristics and outcomes.[127] Instrument choice will be determined by analyses described in section 2.C.7.1. The IV analysis will use two-stage linear regression[128] and will report risk differences adjusted for measured patient factors with robust standard errors.[129] We will also report relative risk measures.[130] Similar to RCTs, we will examine imbalances of measured patient attributes (**Table 3**) between the IV categories at the beginning of treatment using the Mahalanobis distance.[131]

2.C.7.3. Treatment Effect Heterogeneity, Follow-up, and Dose-Response (Aim 3). Models described under 2.C.7.2 will be stratified by age and severity of CV disease (2.C.5.4). Stratum-specific estimates will be reported. Interaction terms will test whether effect modification is significant. In addition, we will estimate average hazard ratios by follow-up period (0-60, 61-180, and 181-365 days). To avoid potential selection bias from stratification of follow-up,[132] we will also estimate hazard ratios for 0-60 and 0-180 days. We will perform dose-response analysis for SGA augmentation episodes combined and for individual SGAs.[46,47] If we find evidence for dose-response, we will also estimate dose-stratified and dose-adjusted hazard ratios.[46]

2.C.8. Project Management and Timeline (Table 5). Dr. Gerhard will assume overall scientific and administrative responsibility for the project and chair monthly calls with Drs. Olfson, Correll and Crystal to review progress and refine the analytic approach. He will also supervise data management and analysis. Data will be maintained and analyzed on the Center's high-security data network. Dr. Huang, a senior programmer experienced with the MAX data, will manage the data and statistical programming.

Table 5: Project Timeline

Task	Q1	Q2	Q3	Q4	Q5	Q6	Q7	Q8
Dataset Construction	X	X						
Variable Construction		X	X	X				
Examine Aim 1				X	X			
Examine Aim 2					X	X	X	
Examine Aim 3							X	X
Report Writing						X	X	X

2.C.9. Limitations and Strengths. The study offers the opportunity to significantly broaden understanding of SGA augmentation safety for depression including new information on individual drugs and treatment safety effect heterogeneity. Although it is subject to some potential limitations, it has several offsetting strengths.

2.C.9.1. Confounding due to Selective Prescribing Practices and its Control. Prescribers and patients make decisions on choice of augmentation strategies in light of patient characteristics and preferences, and clinical experience. When such factors are also risk factors for the safety outcomes, they may bias results. We discuss several methods to minimize this "channeling" (2.C.5.3) and will carefully examine treatment patterns and predictors to further inform confounder control (2.C.7.1). To address residual confounding, we will perform quantitative sensitivity analysis for unmeasured factors such as BMI or family history and explore IV analysis that exploits observed variation in treatment preference at the geographic[118] or prescriber[117] level.

2.C.9.2. Measurement of Exposure and Outcomes. Pharmacy dispensing records are highly accurate.[94-97] Nonetheless, prescription claims data indicate medications dispensed, not ingested. Claims-based outcome definitions may be mis-ascertained since they reflect medical encounters, not structured diagnoses. Yet the measures in 2.C.5.3 demonstrate high specificity or positive predictive values (**Table 2**).[133] All cause mortality data from NDI have demonstrated 100% specificity with sensitivity between 98% and 100%.[36,83,85]

2.C.9.3. Applicability to Usual Care Populations. Our study includes many patients who would not meet enrollment criteria for traditional randomized controlled clinical trials.[134] Although we exclusively examine Medicaid patients, MDD is correlated with lower income,[3] and public insurance accounts for ~40% of all SGA augmentation in depression.[31] The impact of the requirement of continuous Medicaid eligibility on generalizability will be empirically evaluated (2.C.7.1) and taken into account in the reporting of findings.

We will not be able to determine the extent to which observed group differences in premature mortality are mediated by differences in antidepressant effectiveness between SGA and AD augmentation or between individual SGA augmentation strategies.[35,40] Instead, our study will estimate the net differences in safety profiles of the competing pharmacological strategies, inform clinical benefit-risk assessment, and contribute to a better understanding of the specific biological pathways of SGA-associated premature mortality.

2.C.9.4. Study Size. The study will use the 10 most recent available years of MAX data available at no cost through the Rutgers Mental Health CERTs. The unprecedented size of this study facilitates detection of rare, but serious, adverse effects not detectable in MDD RCTs and provides sufficient power for the examination of treatment effect heterogeneity and differences between individual augmentation strategies.

2.C.10. Future Directions. The proposed study will help build or diminish support for resource intensive prospective safety studies of SGA augmentation (large simple trials or registry studies) and inform their design.

1. U.S. Census Bureau. 2010 Census Briefs. Age and Sex Composition: 2010. Age and Sex Composition: 2010. (C2010BR-03) Issued May 2011 (http://www.census.gov/prod/cen2010/briefs/c2010br-03.pdf).
2. Greenberg PE, Kessler RC, Birnbaum HG, et al. The economic burden of depression in the United States: how did it change between 1990 and 2000? *J Clin Psychiatry.* 2003;64(12):1465-1475.
3. Kessler RC, Chiu WT, Demler O, Merikangas KR, Walters EE. Prevalence, severity, and comorbidity of 12-month DSM-IV disorders in the National Comorbidity Survey Replication. *Arch Gen Psychiatry.* 2005;62(6):617-627.
4. Rush AJ, Trivedi MH, Wisniewski SR, et al. Acute and longer-term outcomes in depressed outpatients requiring one or several treatment steps: a STAR*D report. *Am J Psychiatry.* 2006;163(11):1905-1917.
5. American Psychiatric Association (APA). Practice guideline for the treatment of patients with major depressive disorder. 3rd ed. Arlington (VA): American Psychiatric Association (APA); 2010 Oct.
6. Philip NS, Carpenter LL, Tyrka AR, Price LH. Pharmacologic approaches to treatment resistant depression: a re-examination for the modern era. *Expert Opin Pharmacother.* 2010;11(5):709-722.
7. Thase ME. Antidepressant combinations: widely used, but far from empirically validated. *Can J Psychiatry.* 2011;56(6):317-323.
8. Nelson JC, Papakostas GI. Atypical antipsychotic augmentation in major depressive disorder: a meta-analysis of placebo-controlled randomized trials. *Am J Psychiatry.* 2009;166(9):980-991.
9. Khullar A, Chokka P, Fullerton D, McKenna S, Blackamn A: A double-blind, randomized, placebo-controlled study of quetiapine as augmentation therapy to SSRI/SNRI agents in the treatment of non-psychotic unipolar depression with residual symptoms, in American Psychiatric Association 2006 Annual Meeting: New Research Abstracts (Toronto, Canada, May 20– 25, 2006). Washington, DC, APA, 2006.
10. Mattingly G, Ilivicky H, Canale J, Anderson R: Quetiapine combination for treatment-resistant depression, in American Psychiatric Association 2006 Annual Meeting: New Research Abstracts (Toronto, Canada, May 20–25, 2006). Washington, DC, APA, 2006.
11. Bauer M, Pretorius HW, Constant EL, Earley WR, Szamosi J, Brecher M. Extended-release quetiapine as adjunct to an antidepressant in patients with major depressive disorder: results of a randomized, placebo-controlled, double-blind study. *J Clin Psychiatry.* 2009;70(4):540-549.
12. Berman RM, Fava M, Thase ME, et al. Aripiprazole augmentation in major depressive disorder: a double-blind, placebo-controlled study in patients with inadequate response to antidepressants. *CNS Spectr.* 2009;14(4):197-206.
13. Berman RM, Marcus RN, Swanink R, et al. The efficacy and safety of aripiprazole as adjunctive therapy in major depressive disorder: a multicenter, randomized, double-blind, placebo-controlled study. *J Clin Psychiatry.* 2007;68(6):843-853.
14. Corya SA, Williamson D, Sanger TM, Briggs SD, Case M, Tollefson G. A randomized, double-blind comparison of olanzapine/fluoxetine combination, olanzapine, fluoxetine, and venlafaxine in treatment-resistant depression. *Depress Anxiety.* 2006;23(6):364-372.
15. El-Khalili N, Joyce M, Atkinson S, et al. Extended-release quetiapine fumarate (quetiapine XR) as adjunctive therapy in major depressive disorder (MDD) in patients with an inadequate response to ongoing antidepressant treatment: a multicentre, randomized, double-blind, placebo-controlled study. *Int J Neuropsychopharmacol.* 2010;13(7):917-932.
16. Keitner GI, Garlow SJ, Ryan CE, et al. A randomized, placebo-controlled trial of risperidone augmentation for patients with difficult-to-treat unipolar, non-psychotic major depression. *J Psychiatr Res.* 2009;43(3):205-214.
17. Mahmoud RA, Pandina GJ, Turkoz I, et al. Risperidone for treatment-refractory major depressive disorder: a randomized trial. *Ann Intern Med.* 2007;147(9):593-602.
18. Marcus RN, McQuade RD, Carson WH, et al. The efficacy and safety of aripiprazole as adjunctive therapy in major depressive disorder: a second multicenter, randomized, double-blind, placebo-controlled study. *J Clin Psychopharmacol.* 2008;28(2):156-165.
19. McIntyre A, Gendron A. Quetiapine adjunct to selective serotonin reuptake inhibitors or venlafaxine in patients with major depression, comorbid anxiety, and residual depressive symptoms: a randomized, placebo-controlled pilot study. *Depress Anxiety.* 2007;24(7):487-494.
20. Reeves H, Batra S, May RS, Zhang R, Dahl DC, Li X. Efficacy of risperidone augmentation to antidepressants in the management of suicidality in major depressive disorder: a randomized, double-blind, placebo-controlled pilot study. *J Clin Psychiatry.* 2008;69(8):1228-1336.
21. Shelton RC, Tollefson GD, Tohen M, et al. A novel augmentation strategy for treating resistant major depression. *Am J Psychiatry.* 2001;158(1):131-134.

22. Shelton RC, Williamson DJ, Corya SA, et al. Olanzapine/fluoxetine combination for treatment-resistant depression: a controlled study of SSRI and nortriptyline resistance. *J Clin Psychiatry.* 2005;66(10):1289-1297.
23. Thase ME, Corya SA, Osuntokun O, et al. A randomized, double-blind comparison of olanzapine/fluoxetine combination, olanzapine, and fluoxetine in treatment-resistant major depressive disorder. *J Clin Psychiatry.* 2007;68(2):224-236.
24. Spielmans GI, Berman MI, Linardatos E, Rosenlicht NZ, Perry A, Tsai AC. Adjunctive atypical antipsychotic treatment for major depressive disorder: a meta-analysis of depression, quality of life, and safety outcomes. *PLoS medicine.* 2013;10(3):e1001403.
25. Correll CU, Lencz T, Malhotra AK. Antipsychotic drugs and obesity. *Trends Mol Med.* 2011;17(2):97-107.
26. De Hert M, Correll CU, Bobes J, et al. Physical illness in patients with severe mental disorders. I. Prevalence, impact of medications and disparities in health care. *World Psychiatry.* 2011;10(1):52-77.
27. De Hert M, Detraux J, van Winkel R, Yu W, Correll CU. Metabolic and cardiovascular adverse effects associated with antipsychotic drugs. *Nat Rev Endocrinol.* 2011;8(2):114-126.
28. Nielsen J, Skadhede S, Correll CU. Antipsychotics associated with the development of type 2 diabetes in antipsychotic-naive schizophrenia patients. *Neuropsychopharmacology.* 2010;35(9):1997-2004.
29. Ray WA, Chung CP, Murray KT, Hall K, Stein CM. Atypical antipsychotic drugs and the risk of sudden cardiac death. *N Engl J Med.* 2009;360(3):225-235.
30. Schneider LS, Dagerman KS, Insel P. Risk of death with atypical antipsychotic drug treatment for dementia: meta-analysis of randomized placebo-controlled trials. *JAMA.* 2005;294(15):1934-1943.
31. Gerhard T, Akincigil A, Foglio N, Crystal S, Olfson M. National Trends in Antipsychotic Medication Use for Non-Psychotic Depression. (in press, *J Clin Psych;* see appendix).
32. DeRubeis RJ, Gelfand LA, Tang TZ, Simons AD. Medications versus cognitive behavior therapy for severely depressed outpatients: mega-analysis of four randomized comparisons. *Am J Psychiatry.* 1999;156(7):1007-1013.
33. von Wolff A, Holzel LP, Westphal A, Harter M, Kriston L. Combination of pharmacotherapy and psychotherapy in the treatment of chronic depression: a systematic review and meta-analysis. *BMC Psychiatry.*12:61.
34. http://www.nimh.nih.gov/statistics/2LIDD.shtml.
35. Barth J, Schumacher M, Herrmann-Lingen C. Depression as a risk factor for mortality in patients with coronary heart disease: a meta-analysis. *Psychosom Med.* 2004;66(6):802-813.
36. Bostwick JM, Pankratz VS. Affective disorders and suicide risk: a reexamination. *Am J Psychiatry.* 2000;157(12):1925-1932.
37. Broadhead WE, Blazer DG, George LK, Tse CK. Depression, disability days, and days lost from work in a prospective epidemiologic survey. *JAMA.* 1990;264(19):2524-2528.
38. Gump BB, Matthews KA, Eberly LE, Chang YF. Depressive symptoms and mortality in men: results from the Multiple Risk Factor Intervention Trial. *Stroke.* 2005;36(1):98-102.
39. Kessler RC, Berglund P, Demler O, et al. The epidemiology of major depressive disorder: results from the National Comorbidity Survey Replication (NCS-R). *JAMA.* 2003;289(23):3095-3105.
40. Osby U, Brandt L, Correia N, Ekbom A, Sparen P. Excess mortality in bipolar and unipolar disorder in Sweden. *Arch Gen Psychiatry.* 2001;58(9):844-850.
41. Rahman I, Humphreys K, Bennet AM, Ingelsson E, Pedersen NL, Magnusson PK. Clinical depression, antidepressant use and risk of future cardiovascular disease. *European journal of epidemiology.* 2013;28(7):589-595.
42. Pratt LA, Brody DJ, Gu Q. Antidepressant use in persons aged 12 and over: United States, 2005-2008. *NCHS Data Brief.* (76):1-8.
43. Rush AJ, Fava M, Wisniewski SR, et al. Sequenced treatment alternatives to relieve depression (STAR*D): rationale and design. *Control Clin Trials.* 2004;25(1):119-142.
44. Mohamed S, Leslie DL, Rosenheck RA. Use of antipsychotics in the treatment of major depressive disorder in the U.S. Department of Veterans Affairs. *J Clin Psychiatry.* 2009;70(6):906-912.
45. FDA Public Health Advisory: Deaths with Antipsychotics in Elderly Patients with Behavioral Disturbances.[URL:http://www.fda.gov/Drugs/DrugSafety/PostmarketDrugSafetyInformationforPatientsandProviders/DrugSafetyInformationforHeathcareProfessionals/PublicHealthAdvisories/ucm053171.htm. Last accessed January 18, 2013.
46. Gerhard T, Huybrechts K, Olfson M, et al. Comparative mortality risks of antipsychotic medications in community-dwelling older adults. *Br J Psychiatry.* 2013.

47. Huybrechts KF, Gerhard T, Crystal S, et al. Differential risk of death in older residents in nursing homes prescribed specific antipsychotic drugs: population based cohort study. *BMJ.* 2012;344:e977.
48. Haroon E, Raison CL, Miller AH. Psychoneuroimmunology meets neuropsychopharmacology: translational implications of the impact of inflammation on behavior. *Neuropsychopharmacology.* 2012;37(1):137-162.
49. Miller AH, Haroon E, Raison CL, Felger JC. Cytokine targets in the brain: impact on neurotransmitters and neurocircuits. *Depress Anxiety.* 2013;30(4):297-306.
50. Fitzgerald P, O'Brien SM, Scully P, Rijkers K, Scott LV, Dinan TG. Cutaneous glucocorticoid receptor sensitivity and pro-inflammatory cytokine levels in antidepressant-resistant depression. *Psychol Med.* 2006;36(1):37-43.
51. Correll CU, Manu P, Olshanskiy V, Napolitano B, Kane JM, Malhotra AK. Cardiometabolic risk of second-generation antipsychotic medications during first-time use in children and adolescents. *JAMA.* 2009;302(16):1765-1773.
52. Jonsson AK, Spigset O, Hagg S. Venous thromboembolism in recipients of antipsychotics: incidence, mechanisms and management. *CNS Drugs.* 2012;26(8):649-662.
53. Ray WA, Meredith S, Thapa PB, Meador KG, Hall K, Murray KT. Antipsychotics and the risk of sudden cardiac death. *Arch Gen Psychiatry.* 2001;58(12):1161-1167.
54. Setoguchi S, Wang PS, Alan Brookhart M, Canning CF, Kaci L, Schneeweiss S. Potential causes of higher mortality in elderly users of conventional and atypical antipsychotic medications. *J Am Geriatr Soc.* 2008;56(9):1644-1650.
55. Leonard CE, Freeman CP, Newcomb CW, et al. Antipsychotics and the Risks of Sudden Cardiac Death and All-Cause Death: Cohort Studies in Medicaid and Dually-Eligible Medicaid-Medicare Beneficiaries of Five States. *Journal of clinical & experimental cardiology.* 2013;Suppl 10(6):1-9.
56. Stollberger C, Huber JO, Finsterer J. Antipsychotic drugs and QT prolongation. *International clinical psychopharmacology.* 2005;20(5):243-251.
57. Leucht S, Cipriani A, Spineli L, et al. Comparative efficacy and tolerability of 15 antipsychotic drugs in schizophrenia: a multiple-treatments meta-analysis. *Lancet.* 2013;382(9896):951-962.
58. Kales HC, Zivin K, Kim HM, et al. Trends in antipsychotic use in dementia 1999-2007. *Arch Gen Psychiatry.* 2011;68(2):190-197.
59. Dusetzina SB, Higashi AS, Dorsey ER, et al. Impact of FDA drug risk communications on health care utilization and health behaviors: a systematic review. *Med Care.*50(6):466-478.
60. Schneeweiss S, Patrick AR, Sturmer T, et al. Increasing levels of restriction in pharmacoepidemiologic database studies of elderly and comparison with randomized trial results. *Med Care.* 2007;45(10 Supl 2):S131-142.
61. Centers for Medicare and Medicaid Services. Research Data Assistance Center (RESDAC). Data Availability. http://www.resdac.org/cms-data/file-availability. Accessed 11/05/2013.
62. Setoguchi S, Gerhard T. Comparator selection. In: Velentgas P, Dreyer NA, Nourjah P, et al., eds. Developing a Protocol for Observational Comparative Effectiveness Research: A User’s Guide. AHRQ Publication No. 12(13)-EHC099. Rockville, MD: Agency for Healthcare Research and Quality; January 2013: Chapter 5, pp. 59-70. www.effectivehealthcare.ahrq.gov/Methods-OCER.cfm
63. Kales HC, Kim HM, Zivin K, et al. Risk of mortality among individual antipsychotics in patients with dementia. *Am J Psychiatry.* 2012;169(1):71-79.
64. Johnson ES, Bartman BA, Briesacher BA, et al. The incident user design in comparative effectiveness research. *Pharmacoepidemiol Drug Saf.*22(1):1-6.
65. Ray WA. Evaluating medication effects outside of clinical trials: new-user designs. *Am J Epidemiol.* 2003;158(9):915-920.
66. Brauer R, Douglas I, Smeeth L. The association between antipsychotic agents and the risk of myocardial infarction: a systematic review. *Br J Clin Pharmacol.*72(6):871-878.
67. Douglas IJ, Smeeth L. Exposure to antipsychotics and risk of stroke: self controlled case series study. *BMJ.* 2008;337:a1227.
68. Wang S, Linkletter C, Dore D, Mor V, Buka S, Maclure M. Age, antipsychotics, and the risk of ischemic stroke in the Veterans Health Administration. *Stroke.*43(1):28-31.
69. Knol W, van Marum RJ, Jansen PA, Souverein PC, Schobben AF, Egberts AC. Antipsychotic drug use and risk of pneumonia in elderly people. *J Am Geriatr Soc.* 2008;56(4):661-666.
70. Cox DR. Regression models and life tables. *J Roy Statist Soc B* 1972;34:187–220.
71. Arbogast PG, Seeger JD, DEcIDE Methods Center Summary Variable Working Group. Summary Variables in Observational Research: Propensity Scores and Disease Risk Scores. Effective Health Care Program Research Report No. 33. (Prepared by DEcIDE Methods Center under Contract No. HHSA 290-2005-0016-I, Task Order 10.)

AHRQ Publication No. 11(12)-EHC055-EF. Rockville, MD: Agency for Healthcare Research and Quality. May 2012. http://effectivehealthcare.ahrq.gov/reports/final.cfm.
72. Rosenbaum PR, Rubin DB. The central role of the propensity score in observational studies for causal effects. *Biometrika.* 1983;70:41-55.
73. Davis BR, Furberg CD, Williams CB. Survival analysis of adverse effects data in the Beta-Blocker Heart Attack Trial. *Clin Pharmacol Ther.* 1987;41(6):611-615.
74. Braitman LE, Rosenbaum PR. Rare outcomes, common treatments: analytic strategies using propensity scores. *Ann Intern Med.* 2002;137(8):693-695.
75. Cepeda MS, Boston R, Farrar JT, Strom BL. Comparison of logistic regression versus propensity score when the number of events is low and there are multiple confounders. *Am J Epidemiol.* 2003;158(3):280-287.
76. Schneeweiss S. Sensitivity analysis and external adjustment for unmeasured confounders in epidemiologic database studies of therapeutics. *Pharmacoepidemiol Drug Saf.* 2006;15(5):291-303.
77. Angrist JD, Imbens GW, Rubin DB. Identification of causal effects using instrumental variables. *J Am Stat Soc.* 1996;91:444-455.
78. Stroup TS, Gerhard T, Crystal S, Huang C, Olfson M. Geographic and clinical variation in clozapine use in the US. *Psychiatric Services*. Epub ahead of print.
79. Huybrechts KF, Gerhard T, Franklin JM, Levin R, Crystal S, Schneeweiss S. Instrumental Variable Analyses Using Nursing Home Prescribing Preferences for Comparative Effectiveness Research. Pharmacoepidemiology and Drug Safety, 2013;22:S,137. (Manuscript under review).
80. Huybrechts KF, Rothman KJ, Brookhart MA, et al. Variation in antipsychotic treatment choice across US nursing homes. *J Clin Psychopharmacol.* 2012;32(1):11-17.
81. Byrd V, Hedley-Dodd A. Assessing the Usability of Encounter Data for Enrollees in Comprehensive Managed Care Across MAX 2007–2009. Medicaid Policy Brief 15. Mathematica; December 2012.
82. Lewin Group. Evaluating Encounter Data Completeness -- for Researchers Using the CMS Chronic Condition Warehouse. May 9, 2012. Accessed 11/17/13 at https://www.ccwdata.org/cs/groups/public/documents/training/ccw_max_research_example_enctr.pdf.
83. Lash TL, Silliman RA. A comparison of the National Death Index and Social Security Administration databases to ascertain vital status. *Epidemiology.* 2001;12(2):259-261.
84. Patterson BH, Bilgrad R. Use of the National Death Index in cancer studies. *J Natl Cancer Inst.* 1986;77(4):877-881.
85. Rich-Edwards JW, Corsano KA, Stampfer MJ. Test of the National Death Index and Equifax Nationwide Death Search. *Am J Epidemiol.* 1994;140(11):1016-1019.
86. Sathiakumar N, Delzell E, Abdalla O. Using the National Death Index to obtain underlying cause of death codes. *J Occup Environ Med.* 1998;40(9):808-813.
87. Health Resources and Services Administration. Area resource file (ARF) system. In: Bureau of Health Professions, editor. Fairfax, VA: Quality Resource Systems, Inc.; 2006.
88. Frayne SM, Miller DR, Sharkansky EJ, et al. Using administrative data to identify mental illness: what approach is best? *Am J Med Qual.* 2010;25(1):42-50.
89. Smith EG, Henry AD, Zhang J, Hooven F, Banks SM. Antidepressant adequacy and work status among medicaid enrollees with disabilities: a restriction-based, propensity score-adjusted analysis. *Community Ment Health J.* 2009;45(5):333-340.
90. Solberg LI, Engebretson KI, Sperl-Hillen JM, Hroscikoski MC, O'Connor PJ. Are claims data accurate enough to identify patients for performance measures or quality improvement? The case of diabetes, heart disease, and depression. *Am J Med Qual.* 2006;21(4):238-245.
91. Fava M. Diagnosis and definition of treatment-resistant depression. *Biol Psychiatry.* 2003;53(8):649-659.
92. Sackeim HA. The definition and meaning of treatment-resistant depression. *J Clin Psychiatry.* 2001;62 Suppl 16:10-17.
93. Thase ME, Rush AJ. When at first you don't succeed: sequential strategies for antidepressant nonresponders. *J Clin Psychiatry.* 1997;58 Suppl 13:23-29.
94. Crystal S, Akincigil A, Bilder S, Walkup JT. Studying prescription drug use and outcomes with medicaid claims data: strengths, limitations, and strategies. *Med Care.* 2007;45(10 Supl 2):S58-65.
95. Levy AR, O'Brien BJ, Sellors C, Grootendorst P, Willison D. Coding accuracy of administrative drug claims in the Ontario Drug Benefit database. *Can J Clin Pharmacol.* 2003;10(2):67-71.
96. Ray WA, Griffin MR. Use of Medicaid data for pharmacoepidemiology. *Am J Epidemiol.* 1989;129(4):837-849.

97. Stergachis AS. Record linkage studies for postmarketing drug surveillance: data quality and validity considerations. *Drug Intell Clin Pharm.* 1988;22(2):157-161.
98. Valuck RJ, Orton HD, Libby AM. Antidepressant discontinuation and risk of suicide attempt: a retrospective, nested case-control study. *J Clin Psychiatry.* 2009;70(8):1069-1077.
99. Andreasen NC, Pressler M, Nopoulos P, Miller D, Ho BC. Antipsychotic dose equivalents and dose-years: a standardized method for comparing exposure to different drugs. *Biol Psychiatry.*67(3):255-262.
100. Chung CP, Murray KT, Stein CM, Hall K, Ray WA. A computer case definition for sudden cardiac death. *Pharmacoepidemiol Drug Saf.*19(6):563-572.
101. Austin PC, Daly PA, Tu JV. A multicenter study of the coding accuracy of hospital discharge administrative data for patients admitted to cardiac care units in Ontario. *Am Heart J.* 2002;144(2):290-296.
102. Choma NN, Griffin MR, Huang RL, et al. An algorithm to identify incident myocardial infarction using Medicaid data. *Pharmacoepidemiol Drug Saf.* 2009;18(11):1064-1071.
103. Kiyota Y, Schneeweiss S, Glynn RJ, Cannuscio CC, Avorn J, Solomon DH. Accuracy of Medicare claims-based diagnosis of acute myocardial infarction: estimating positive predictive value on the basis of review of hospital records. *Am Heart J.* 2004;148(1):99-104.
104. Levy AR, Tamblyn RM, Fitchett D, McLeod PJ, Hanley JA. Coding accuracy of hospital discharge data for elderly survivors of myocardial infarction. *Can J Cardiol.* 1999;15(11):1277-1282.
105. Petersen LA, Wright S, Normand SL, Daley J. Positive predictive value of the diagnosis of acute myocardial infarction in an administrative database. *J Gen Intern Med.* 1999;14(9):555-558.
106. Kokotailo RA, Hill MD. Coding of stroke and stroke risk factors using international classification of diseases, revisions 9 and 10. *Stroke.* 2005;36(8):1776-1781.
107. Roumie CL, Mitchel E, Gideon PS, Varas-Lorenzo C, Castellsague J, Griffin MR. Validation of ICD-9 codes with a high positive predictive value for incident strokes resulting in hospitalization using Medicaid health data. *Pharmacoepidemiol Drug Saf.* 2008;17(1):20-26.
108. Tirschwell DL, Longstreth WT, Jr. Validating administrative data in stroke research. *Stroke.* 2002;33(10):2465-2470.
109. Hebert PL, Geiss LS, Tierney EF, Engelgau MM, Yawn BP, McBean AM. Identifying persons with diabetes using Medicare claims data. *Am J Med Qual.* 1999;14(6):270-277.
110. Rector TS, Wickstrom SL, Shah M, et al. Specificity and sensitivity of claims-based algorithms for identifying members of Medicare+Choice health plans that have chronic medical conditions. *Health Serv Res.* 2004;39(6 Pt 1):1839-1857.
111. Willey VJ, Bullano MF, Hauch O, et al. Management patterns and outcomes of patients with venous thromboembolism in the usual community practice setting. *Clin Ther.* 2004;26(7):1149-1159.
112. Glynn RJ, Knight EL, Levin R, Avorn J. Paradoxical relations of drug treatment with mortality in older persons. *Epidemiology.* 2001;12(6):682-689.
113. Redelmeier DA, Tan SH, Booth GL. The treatment of unrelated disorders in patients with chronic medical diseases. *The New England journal of medicine.* 1998;338(21):1516-1520.
114. Oquendo MA, Baca-Garcia E, Kartachov A, et al. A computer algorithm for calculating the adequacy of antidepressant treatment in unipolar and bipolar depression. *J Clin Psychiatry.* 2003;64(7):825-833.
115. Mamdani M, Sykora K, Li P, et al. Reader's guide to critical appraisal of cohort studies: 2. Assessing potential for confounding. *BMJ.* 2005;330(7497):960-962.
116. Lehman AF, Kreyenbuhl J, Buchanan RW, et al. The Schizophrenia Patient Outcomes Research Team (PORT): updated treatment recommendations 2003. *Schizophr Bull.* 2004;30(2):193-217.
117. Brookhart MA, Wang PS, Solomon DH, Schneeweiss S. Evaluating short-term drug effects using a physician-specific prescribing preference as an instrumental variable. *Epidemiology.* 2006;17(3):268-275.
118. Stukel TA, Fisher ES, Wennberg DE, Alter DA, Gottlieb DJ, Vermeulen MJ. Analysis of observational studies in the presence of treatment selection bias: effects of invasive cardiac management on AMI survival using propensity score and instrumental variable methods. *JAMA.* 2007;297(3):278-285.
119. Kaplan EL, Meier P. Nonparametric estimation from incomplete observations. J Am Stat Ass 1958;53:457-81.
120. Cooper-DeHoff RM, Bird ST, Nichols GA, Delaney JC, Winterstein AG. Association of antihypertensive drug classes, alone or in combination with incident diabetes: a nested case-control study. Circulation 2012;126:A11073.

121. Sturmer T, Rothman KJ, Avorn J, Glynn RJ. Treatment effects in the presence of unmeasured confounding: dealing with observations in the tails of the propensity score distribution--a simulation study. *Am J Epidemiol.* 2010;172(7):843-854.
122. Mack CD, Glynn RJ, Brookhart MA, et al. Calendar time-specific propensity scores and comparative effectiveness research for stage III colon cancer chemotherapy. *Pharmacoepidemiol Drug Saf.* 2013;22(8):810-818.
123. Bowden RJ, Turkington DA. *Instrumental variables.* Cambridge Cambridgeshire ; New York: Cambridge University Press; 1984.
124. Brookhart MA, Rassen JA, Schneeweiss S. Instrumental variable methods in comparative safety and effectiveness research. *Pharmacoepidemiol Drug Saf.* 2010;19(6):537-554.
125. Angrist JD, Krueger AB. Instrumental variables and the search for identification: from supply and demand to natural experiments. *J Econ Perspectives.* 2001;15:69-85.
126. McClellan M, McNeil BJ, Newhouse JP. Does more intensive treatment of acute myocardial infarction in the elderly reduce mortality? Analysis using instrumental variables. *JAMA.* 1994;272(11):859-866.
127. Greenland S. An introduction to instrumental variables for epidemiologists. *Int J Epidemiol.* 2000;29(4):722-729.
128. Greene WH. Econometric analysis. Upper Saddle River, N.J.: Prentice Hall; 1997.
129. Zeger SL, Liang KY. Longitudinal data analysis for discrete and continuous outcomes. *Biometrics.* 1986;42(1):121-130.
130. Rassen JA, Brookhart MA, Glynn RJ, Mittleman MA, Schneeweiss S. Instrumental variables I: instrumental variables exploit natural variation in nonexperimental data to estimate causal relationships. *J Clin Epidemiol.* 2009;62(12):1226-1232.
131. Schneeweiss S, Solomon DH, Wang PS, Rassen J, Brookhart MA. Simultaneous assessment of short-term gastrointestinal benefits and cardiovascular risks of selective cyclooxygenase 2 inhibitors and nonselective nonsteroidal antiinflammatory drugs: an instrumental variable analysis. *Arthritis Rheum.* 2006;54(11):3390-3398.
132. Hernan MA. The hazards of hazard ratios. *Epidemiology.*21(1):13-15.
133. Kelsey JL, Whittemore AS, Evans AS, Thompson WD. M*ethods in observational epidemiology,* 2nd edition. New York: Oxford University Press;1996:p.341-390.
134. Blanco C, Olfson M, Goodwin RD, et al. Generalizability of clinical trial results for major depression to community samples: results from the National Epidemiologic Survey on Alcohol and Related Conditions. *J Clin Psychiatry.* 2008;69(8):1276-1280.
